# Associating Multivariate Traits with Genetic Variants Using Collapsing and Kernel Methods with Pedigree- or Population-Based Studies

**DOI:** 10.1155/2021/8812282

**Published:** 2021-02-09

**Authors:** Li-Chu Chien

**Affiliations:** Center for Fundamental Science, Kaohsiung Medical University, Kaohsiung, Taiwan

## Abstract

In genetic association analysis, several relevant phenotypes or multivariate traits with different types of components are usually collected to study complex or multifactorial diseases. Over the past few years, jointly testing for association between multivariate traits and multiple genetic variants has become more popular because it can increase statistical power to identify causal genes in pedigree- or population-based studies. However, most of the existing methods mainly focus on testing genetic variants associated with multiple continuous phenotypes. In this investigation, we develop a framework for identifying the pleiotropic effects of genetic variants on multivariate traits by using collapsing and kernel methods with pedigree- or population-structured data. The proposed framework is applicable to the burden test, the kernel test, and the omnibus test for autosomes and the X chromosome. The proposed multivariate trait association methods can accommodate continuous phenotypes or binary phenotypes and further can adjust for covariates. Simulation studies show that the performance of our methods is satisfactory with respect to the empirical type I error rates and power rates in comparison with the existing methods.

## 1. Introduction

Genome-wide association studies (GWAS) intend to find genetic variants such as single nucleotide polymorphisms (SNPs) associated with common traits or with complex diseases [1, 2]. Association studies, where the correlation relationship between a genetic variant and a trait is evaluated, are helpful for mapping genes influencing complex diseases [3]. In the study of complex diseases, data on several correlated phenotypes or a multivariate phenotype with several components are often collected to get a better understanding of the disease [1, 3, 4]. Multivariate correlated traits are influenced through multiple variants simultaneously. Therefore, by a suitable joint or multivariate analysis framework of multivariate traits, we can not only gain more statistical power to identify pleiotropic effects of genetic variants on multivariate traits [3, 5–12] but also can further understand the genetic architecture of the disease of interest [5, 13]. Thus, recently, the joint analysis of multivariate traits has become popular because it can increase statistical power over analyzing only one trait at a time [1, 4].

Several statistical methods have been developed to identify the association between multivariate traits and a genetic variant [1, 5]. Current multivariate methods can be classified into three groups [1, 2, 5]: regression methods [14–16], variable reduction methods [11, 13, 17, 18], and combining analysis [9, 19–23]. However, many of the existing methods for multivariate association analysis cannot be straightaway extended to rare variant analyses, due to their enormous numbers causing the problems of multiple comparison or multiple testing and their low minor allele frequencies [2, 5, 24]. Moreover, sparsity of data could lead to problems on estimating regression parameters and fitting regression models [2]. Hence, it is necessary for proposing statistical methods for identifying the association between multivariate traits and multiple genetic variants (common and/or rare variants) [5]. In recent years, various statistical techniques have been proposed for this purpose in GWAS [8, 17, 25–27]. Furthermore, several approaches have been extendedly developed for the investigation of rare variants associated with multivariate traits [2, 28–38].

Although these new developments keep many benefits, existing methods have some potential limitations [39]. Most current methods are constructed under some specific assumptions about the effects of genetic variants on multivariate traits [39]. These current approaches suffer a severe loss in power once the model assumptions are violated [26, 39].

In this investigation, we develop the statistical methods for identifying pleiotropic effects of genetic variants on multivariate traits using collapsing and kernel methods with pedigree- or population-structured data. The proposed multivariate trait association method is able to handle binary phenotypes or continuous phenotypes and further can adjust for covariates. Moreover, the proposed multivariate trait association method not only can leverage the dependence on the phenotypes but also can account for the sample relatedness in the pedigree-based or population-based structured data.

The rest of the article is organized as follows. In the materials and methods section, we construct the multivariate effect model using the joint GEE model formulation (JGEE) [40]. We apply the JGEE to pedigree- or population-structured data and introduce a retrospective framework for analyzing multivariate traits in genetic association studies. The proposed framework is applicable to the burden test, the kernel test, and the omnibus test for autosomes and the X chromosome. In the simulation studies, we examine the finite sample size performance of the proposed multivariate association methods and evaluate the comparison results with the existing method, Multi-SKAT [39]. Concluding remarks and future possibilities for continuity are given in the conclusion section and the limitation section.

## 2. Materials and Methods

### 2.1. Notations

To describe the proposed multivariate trait association method based on the pedigree- or population-based structured data, we suppose that there are *N* independent pedigrees and each pedigree has *n*_*i*_ subjects. We assume that the *n*_*i*_ subjects have been sequenced in a genetic region of interest (e.g., a gene) that contains *p* variants. Let **y**_*ik*_ = (*y*_*i*1*k*_, *y*_*i*2*k*_,⋯,*y*_in_*i*_*k*_)^*T*^ be the *n*_*i*_ × 1 phenotype vector for the *k*^th^ phenotype of the *i*^th^ pedigree. Let **y**_*i*_ = (**y**_*i*1_, **y**_*i*2_, ⋯, **y**_*iK*_) be the (*n*_*i*_ × *K*) × 1 response vector for the *K* phenotypes that we are interested in. Let **x**_*im*_ = (*x*_*i*1*m*_, *x*_*i*2*m*_,⋯,*x*_in_*i*_*m*_)^*T*^ be the *n*_*i*_ × 1 vector for the *m*^th^ covariate of the *i*^th^ pedigree. Let **x**_*i*_ = (**x**_*i*0_, **x**_*i*1_, ⋯, **x**_*iq*_) be the *n*_*i*_ × (*q* + 1) covariate matrix for the (*q* + 1) nongenetic covariates that we want to adjust for. Let **α**_*k*_ = (*α*_0*k*_, *α*_1*k*_,⋯,*α*_*qk*_)^*T*^ be the (*q* + 1) × 1 vector of regression coefficients of the (*q* + 1) nongenetic covariates with the element *α*_*mk*_ being the effect of the *m*^th^ covariate on the *k*^th^ trait. Let **g**_*i*_ = (**g**_*i*1_, **g**_*i*2_, ⋯, **g**_*ip*_) be the *n*_*i*_ × *p* genetic matrix for *p* genetic variants in a target region of interest where **g**_*il*_ = (g_*i*1*l*_, g_*i*2*l*_,⋯,g_in_*i*_*l*_)^*T*^ is the *n*_*i*_ × 1 vector for a genetic variant *l* (g_*ijl*_ = 0, 1, or 2 for 0, 1, or 2 copies of the minor allele, respectively). Let **β**_*k*_ = (*β*_1*k*_, *β*_2*k*_,⋯,*β*_*pk*_)^*T*^ be the *p* × 1 vector of regression coefficients of the *p* genetic variants with the element *β*_*lk*_ being the effect of the *l*^th^ genetic variant on the *k*^th^ trait.

### 2.2. Multitrait Regression-Based Tests for Pedigree Data

We let **X**_*i*_ = **I**_*K*_ ⊗ **x**_*i*_ be the (*n*_*i*_ × *K*) × ((*q* + 1) × *K*) covariate matrix and **G**_*i*_ = **I**_*K*_ ⊗ **g**_*i*_ be the (*n*_*i*_ × *K*) × (*p* × *K*) genotype matrix for the *i*^th^ pedigree where **I**_*K*_ is an identity matrix of dimension *K* × *K* and ⊗ stands for the Kronecker product. According to the generalized linear model [41], we assume that the marginal density of *y*_*ijk*_ is *f*(*y*_*ijk*_) = exp[{*y*_*ijk*_*θ*_*ijk*_ − *a*(*θ*_*ijk*_) + *b*(*y*_*ijk*_)}/*ϕ*] with two moments, *μ*_*ijk*_ = *E*(*y*_*ijk*_) = *∂a*(*θ*_*ijk*_)/*∂θ*_*ijk*_ and Var(*y*_*ijk*_) = (*∂μ*_*ijk*_/*∂θ*_*ijk*_)*ϕ*, where *ϕ* is a scale parameter. Let **θ**_*i*_ = (**θ**_*i*1_^*T*^, **θ**_*i*2_^*T*^,⋯,**θ**_*iK*_^*T*^)^*T*^ be the (*n*_*i*_ × *K*) × 1 vector with the components **θ**_*ik*_ = (*θ*_*i*1*k*_, *θ*_*i*2*k*_,⋯,*θ*_in_*i*_*k*_)^*T*^, *k* = 1, 2, ⋯, *K* and **η**_*i*_ = (**η**_*i*1_^*T*^, **η**_*i*2_^*T*^,⋯,**η**_*iK*_^*T*^)^*T*^ be the (*n*_*i*_ × *K*) × 1 vector with the components **η**_*ik*_ = (*η*_*i*1*k*_, *η*_*i*2*k*_,⋯,*η*_in_*i*_*k*_)^*T*^ = **x**_*i*_**α**_*k*_ + **g**_*i*_**β**_*k*_, *k* = 1, 2, ⋯, *K* for the *k*^th^ trait of the *i*^th^ pedigree.

Based on the joint GEE model formulation [40], we construct the multivariate linear model for describing the association relationship between *K* correlated traits and genetic variants, which is given as follows:

(1) $$\begin{array}{c} \mu_{i}=g\left(\eta_{i}\right)\text{ and }\eta_{i}=X_{i}\alpha +G_{i}\beta , \end{array}$$

where *g*^−1^(•) is the inverse function of *g*(•) and is a response-specific link function [40], **μ**_*i*_ = (**μ**_*i*1_^*T*^, **μ**_*i*2_^*T*^,⋯,**μ**_*iK*_^*T*^)^*T*^ is the (*n*_*i*_ × *K*) × 1 vector of the expected mean of the multivariate traits **y**_*i*_ = (**y**_*i*1_^*T*^, **y**_*i*2_^*T*^,⋯,**y**_*iK*_^*T*^)^*T*^, **α** = (**α**_1_^*T*^, **α**_2_^*T*^,⋯,**α**_*K*_^*T*^)^*T*^ is the ((*q* + 1) × *K*) × 1 vector of regression coefficients of the (*q* + 1) nongenetic covariates for the *K* correlated traits, and **β** = (**β**_1_^*T*^, **β**_2_^*T*^,⋯,**β**_*K*_^*T*^)^*T*^ is the (*p* × *K*) × 1 vector of regression coefficients of the *p* genetic variants for the *K* correlated traits.

Let **R**_*n*_*i*__(**φ**) and **R**_*K*_(**γ**) be the *n*_*i*_ × *n*_*i*_ within-in cluster correlation matrix and the *K* × *K* multivariate-response cluster correlation matrix, which depend on a vector of parameters **φ** and **γ**, respectively. The (*n*_*i*_ × *K*) × (*n*_*i*_ × *K*) working (or approximate) covariance matrix of **y**_*i*_ is given by [40].

(2) $$\begin{array}{c} V_{i}=A_{i}^{1/2}\left(R_{K}\left(\gamma \right)⊗R_{n_{i}}\left(\varphi \right)\right)A_{i}^{1/2}\phi , \end{array}$$

where **A**_*i*_ = diag(**A**_*i*1_, **A**_*i*2_, ⋯, **A**_*iK*_) is a (*n*_*i*_ × *K*) × (*n*_*i*_ × *K*) block diagonal matrix with the components **A**_*ik*_ = diag(*∂μ*_*i*1*k*_/*∂θ*_*i*1*k*_, *∂μ*_*i*2*k*_/*∂θ*_*i*2*k*_, ⋯, *∂μ*_in_*i*_*k*_/*∂θ*_in_*i*_*k*_), *k* = 1, 2, ⋯, *K* being the *n*_*i*_ × *n*_*i*_ diagonal matrices. According to equation (1), under the null hypothesis of no association between genotypes and phenotypes, we propose the multivariate association methods including the homogeneous kernel statistic (HoK), the heterogeneous kernel statistic (HeK), and burden test (BT). Moreover, we propose the homogeneous omnibus test (HoO) and heterogeneous omnibus test (HeO) by combining the HoK with the BT and by combining the HeK with the BT, respectively.

#### 2.2.1. Kernel Statistic

We let **H** be a *p* × *p* correlation matrix of genotype scores with element *H*_*ll*′_ for markers *l* and *l*′. Let *m*_*l*_ denote the minor allele frequency (MAF) of the *l*^th^ marker. Let $S_{i}={\hat{V}}_{i}^{-1}\left(y_{i}-{\hat{\mu }}_{i}\right)={\left(S_{i1}^{T},S_{i2}^{T},\cdots ,S_{iK}^{T}\right)}^{T}$ be the ((*n*_*i*_ × *K*) × 1) vector of the standard residuals with components **S**_*ik*_ = (*S*_*i*1*k*_, *S*_*i*2*k*_,⋯,*S*_in_*i*_*k*_)^*T*^, *k* = 1, 2, ⋯, *K*, where ${\hat{V}}_{i}^{-1}$ is the inverse matrix of ${\hat{V}}_{i}$. Here, ${\hat{V}}_{i}$ and ${\hat{\mu }}_{i}$ are the estimates of **V**_*i*_ and **μ**_*i*_. Here and henceforth, all estimates are calculated based on the null hypothesis of the genetic effects **β** equal to zero. All unknown parameters and the working within-in and multivariate-response cluster correlation matrices are estimated by the R package JGEE [42].

*(1) Homogeneous Kernel Statistic*. We suppose that *w*_*l*_ is a marker-specific weight of the *l*^th^ variant and assume that the genetic effects on the *K* different phenotypes are homogeneous (i.e., **β**_1_ = **β**_2_ = ⋯ = **β**_*K*_). Based on the JGEE model with the genotype as random variables considered, we propose the homogeneous quadratic (kernel) association statistic (HoK) as follows:

(3) $$\begin{array}{c} \kappa_{\text{Ho}}=\overset{p}{\underset{l=1}{\sum }}{\left[w_{l}\overset{K}{\underset{k=1}{\sum }}\overset{N}{\underset{i=1}{\sum }}g_{il}^{T}{\overset{\wedge }{\Delta }}_{ik}{\overset{\wedge }{A}}_{ik}S_{ik}\right]}^{2}=\overset{p}{\underset{l=1}{\sum }}{\left[w_{l}\overset{K}{\underset{k=1}{\sum }}Z_{lk}\right]}^{2}=\overset{p}{\underset{l=1}{\sum }}{\left[w_{l}Z_{l}\right]}^{2}=\overset{p}{\underset{l=1}{\sum }}{\tilde{Z}}_{l}^{2}, \end{array}$$

where ${\tilde{Z}}_{l}=w_{l}Z_{l}$, *Z*_*l*_ = ∑_*k*=1_^*K*^*Z*_*lk*_, $Z_{lk}=\sum_{i=1}^{N}g_{il}^{T}{\hat{\Delta }}_{ik}{\hat{A}}_{ik}S_{ik}$_,_${\hat{A}}_{ik}$ is the estimate of **A**_*ik*_, and ${\hat{\Delta }}_{ik}$ is the estimate of Δ_*ik*_ = diag(*∂θ*_*i*1*k*_/*∂η*_*i*1*k*_, *∂θ*_*i*2*k*_/*∂η*_*i*2*k*_, ⋯, *∂θ*_in_*i*_*k*_/*∂η*_in_*i*_*k*_) that is a *n*_*i*_ × *n*_*i*_ diagonal matrix for the *k*^th^ phenotype of the *i*^th^ pedigree. The null distribution of *κ*_Ho_ asymptotically follows a mixture chi-square distribution ∑_*l*=1_^*p*^*λ*_*l*_*χ*_*l*,1_^2^, where *χ*_*l*,1_^2^s are independent random variables following a chi-square distribution with one degree of freedom and (*λ*_1_, *λ*_2_, ⋯, *λ*_*p*_) are nonzero eigenvalues of the null covariate matrix of ${\text{Cov}}_{0}\left({\tilde{Z}}_{l},{\tilde{Z}}_{l^{\prime }}\right)=2C_{\text{Ho}}w_{l}w_{l^{\prime }}H_{ll^{\prime }}\sqrt{m_{l}\left(1-m_{l}\right)m_{l^{\prime }}\left(1-m_{l^{\prime }}\right)}$ where $C_{\text{Ho}}=\sum_{i=1}^{N}\left[\left(\sum_{k=1}^{K}S_{ik}^{T}{\hat{A}}_{ik}{\hat{\Delta }}_{ik}\right)\Omega_{i}\left(\sum_{k=1}^{K}{\hat{\Delta }}_{ik}{\hat{A}}_{ik}S_{ik}\right)\right]$ and *Ω*_*i*_ is a *n*_*i*_ × *n*_*i*_ matrix of genetic correlations for all *n*_*i*_ individuals in the *i*^th^ pedigree, which has the same definition given by Schaid et al. [43] and can be calculated by the R package kinship2 [44]. When the genetic relationship between subjects *j* and *j*′ in the *i*^th^ pedigree is unknown, the elements of the genetic correlation *Ω*_*i*_ can be estimated through genomic data [43, 45], and its estimate is given by [43]

(4) $$\begin{array}{c} {\hat{\Omega }}_{i}=\frac{1}{p}\overset{p}{\underset{l=1}{\sum }}\frac{\left({\text{g}}_{ijl}-2m_{l}\right)\left({\text{g}}_{ij^{\prime }l}-2m_{l}\right)}{2m_{l}\left(1-m_{l}\right)}. \end{array}$$

*(2) Heterogeneous Kernel Statistic*. We assume that the genetic effects on the *K* different phenotypes are heterogeneous (i.e., **β**_1_ ≠ **β**_2_≠⋯≠**β**_*K*_). The heterogeneous quadratic (kernel) association statistic (HeK) is defined by

(5) $$\begin{array}{c} \kappa_{\text{He}}=\overset{p}{\underset{l=1}{\sum }}\overset{K}{\underset{k=1}{\sum }}{\left[w_{lk}\overset{N}{\underset{i=1}{\sum }}g_{il}^{T}{\overset{\wedge }{\Delta }}_{ik}{\overset{\wedge }{A}}_{ik}S_{ik}\right]}^{2}=\overset{p}{\underset{l=1}{\sum }}\overset{K}{\underset{k=1}{\sum }}{\left[w_{lk}Z_{lk}\right]}^{2}=\overset{p}{\underset{l=1}{\sum }}\overset{K}{\underset{k=1}{\sum }}{\tilde{Z}}_{lk}^{2}, \end{array}$$

where ${\tilde{Z}}_{lk}=w_{lk}Z_{lk}$ and *w*_*lk*_ is a marker-specific weight of the *l*^th^ variant of the *k*^th^ trait. The null distribution of *κ*_He_ asymptotically follows a mixture chi-square distribution ∑_*l*=1_^(*p* × *K*)^*λ*_*l*_*χ*_*l*,1_^2^, where *χ*_*l*,1_^2^s are independent random variables following a chi-square distribution with one degree of freedom, and (*λ*_1_, *λ*_2_, ⋯, *λ*_(*p* × *K*)_) are nonzero eigenvalues of the null covariate matrix of ${\text{Cov}}_{0}\left({\tilde{\text{Z}}}_{lk},{\tilde{\text{Z}}}_{l^{\prime }k^{\prime }}\right)=2C_{\text{He}}w_{lk}w_{l^{\prime }k^{\prime }}H_{ll^{\prime }}\sqrt{m_{l}\left(1-m_{l}\right)m_{l^{\prime }}\left(1-m_{l^{\prime }}\right)}$, where $C_{\text{He}}=\sum_{i=1}^{N}\left[S_{ik}^{T}{\hat{A}}_{ik}{\hat{\Delta }}_{ik}\Omega_{i}{\hat{\Delta }}_{ik^{\prime }}{\hat{A}}_{ik^{\prime }}S_{ik^{\prime }}\right]$.

Theoretical *p* values of *κ*_Ho_ and *κ*_He_ are approximately calculated by Kuonen's saddlepoint method [46] and obtained by the R package pchisqsum. A theory for the derivation of the HoK test (*κ*_Ho_) and the HeK test (*κ*_He_) is shown in Appendix S1.

#### 2.2.2. Burden Test

We let ${\tilde{g}}_{i}^{T}=\sum_{l=1}^{p}w_{l}g_{il}^{T}$ be a weighted average of genotype scores for the *i*^th^ pedigree. On the basis of the HoK test (*κ*_Ho_) and the HeK test (*κ*_He_) in equations (3) and (5) with the same marker-specific weight of the *l*^th^ variant for each trait *k* (i.e., *w*_*l*_ = *w*_*lk*_, *k* = 1, 2, ⋯, *K*), we propose the burden test (BT) as follows:

(6) $$\begin{array}{c} \text{BT}=\frac{{\left[\sum_{i=1}^{N}\left(\sum_{k=1}^{K}S_{ik}^{T}{\overset{\wedge }{A}}_{ik}{\overset{\wedge }{\Delta }}_{ik}\right){\tilde{g}}_{i}\right]}^{2}}{\sum_{i=1}^{N}\left[\left(\sum_{k=1}^{K}S_{ik}^{T}{\hat{A}}_{ik}{\hat{\Delta }}_{ik}\right){\text{Cov}}_{0}\left({\tilde{g}}_{i}\right)\left(\sum_{k=1}^{K}{\hat{\Delta }}_{ik}{\hat{A}}_{ik}S_{ik}\right)\right]}, \end{array}$$

where the null covariance matrix of ${\tilde{g}}_{i}$ is given by

(7) $$\begin{array}{c} {\text{Cov}}_{0}\left({\tilde{g}}_{i}\right)={\text{Cov}}_{0}\left(\overset{p}{\underset{l=1}{\sum }}w_{l}g_{il}\right)=\overset{p}{\underset{l=1}{\sum }}w_{l}^{2}{\text{Cov}}_{0}\left(g_{il},g_{il}\right)+2\overset{p}{\underset{l=1}{\sum }}\overset{p}{\underset{l^{\prime }=l+1}{\sum }}w_{l}w_{l^{\prime }}{\text{Cov}}_{0}\left(g_{il},g_{il^{\prime }}\right)=\Omega_{i}\overset{p}{\underset{l=1}{\sum }}\overset{p}{\underset{l^{\prime }=1}{\sum }}2w_{l}w_{l^{\prime }}H_{ll^{\prime }}\sqrt{m_{l}\left(1-m_{l}\right)m_{l^{\prime }}\left(1-m_{l^{\prime }}\right)}. \end{array}$$

Then,

(8) $$\begin{array}{c} \text{BT}=\frac{{\left[\sum_{i=1}^{N}\left(\sum_{k=1}^{K}S_{ik}^{T}{\overset{\wedge }{A}}_{ik}{\overset{\wedge }{\Delta }}_{ik}\right){\tilde{g}}_{i}\right]}^{2}}{2\sum_{l=1}^{p}\sum_{l^{\prime }=1}^{p}w_{l}w_{l^{\prime }}H_{ll^{\prime }}\sqrt{m_{l}\left(1-m_{l}\right)m_{l^{\prime }}\left(1-m_{l^{\prime }}\right)}C_{\text{Ho}}}. \end{array}$$

The null distribution of BT asymptotically follows a chi-square distribution with one degree of freedom.

#### 2.2.3. Omnibus Test

Let *p*_Ho_, *p*_He_, and *p*_BT_ denote the *p* values obtained by the HoK, HeK, and BT statistics. Based on the idea of the *p* value combination method through the Cauchy distribution [47–49], we propose the homogeneous omnibus test (HoO) and heterogeneous omnibus test (HeO).

*(1) Homogeneous Omnibus Test*. Combining the *p*_Ho_ with the *p*_BT_, we construct the homogeneous omnibus test (HoO) as follows:

(9) $$\begin{array}{c} O_{\text{Ho}}=-\frac{1}{2}\left[F_{C}^{-1}\left(p_{\text{Ho}}\right)+F_{C}^{-1}\left(p_{\text{BT}}\right)\right], \end{array}$$

where *F*_*C*_^−1^ stands for the inverse cumulative distribution function of the standard Cauchy distribution.

*(2) Heterogeneous Omnibus Test*. Combining the *p*_He_ with the *p*_BT_, we construct the heterogeneous omnibus test (HeO) as follows:

(10) $$\begin{array}{c} O_{\text{He}}=-\frac{1}{2}\left[F_{C}^{-1}\left(p_{\text{He}}\right)+F_{C}^{-1}\left(p_{\text{BT}}\right)\right]. \end{array}$$

The null distributions of the *O*_Ho_ test and the *O*_He_ test asymptotically follow a standard Cauchy distribution [47–49]. The *p* values of the *O*_Ho_ test and the *O*_He_ test are calculated by the R package RNOmni [50].

The kernel statistic, the burden test, and the omnibus test are also applicable to the X chromosome. Additional technical information for extensions to the X chromosome is shown in Appendix S2.

## 3. Simulation Studies

We conduct the numerical simulation studies to assess the finite sample performance of the proposed methods and evaluate the comparison results with two existing methods, the minimum *p* value SKAT statistic (mPK), and the minimum *p* value burden statistic (mPB) [39]. The two existing methods are implemented by the R package Multi-SKAT [39]. Based on the similar simulation set-up as those usually considered from existing genetic association tests [39, 43, 51], we investigate the effect of the proposed methods, HoK, HeK, BT, HoO, and HeO, for identifying genetic variants that are associated with multiple traits. We simultaneously generate 10,000 European-like (EUR) and 10,000 admixed African American-like (AA) haplotypes of length 200 kb using a calibrated human demographic model through the COSI software [51, 52]. A 3 kb region is randomly selected in our numerical simulations. We generate a total of 10,000 databases for each simulation scenario in our studies.

### 3.1. Type I Error Rate and Power Simulations

In the heterogeneous population with nuclear family data considered, continuous and binary phenotypes for trait *k* for individual *j* in the *i*^th^ family are generated from the multivariate linear model in equation (1) with *K* = 2 and *n*_*i*_ = 3. More precisely, continuous and binary phenotypes are generated by the following linear and logit models, respectively:

(11) $$\begin{array}{c} y_{i}=X_{i}\alpha +G_{i}\beta +\varepsilon_{i}, \end{array}$$

(12) $$\begin{array}{c} \text{logit}\left(P\left(y_{i}=1\right)\right)=X_{i}\alpha +G_{i}\beta , \end{array}$$

where **y**_*i*_ = (**y**_*i*1_^*T*^, **y**_*i*2_^*T*^)^*T*^, **X**_*i*_ = **I**_2_ ⊗ **x**_*i*_, **G**_*i*_ = **I**_2_ ⊗ **g**_*i*_, **α** = (**α**_1_^*T*^, **α**_2_^*T*^)^*T*^, **β** = (**β**_1_^*T*^, **β**_2_^*T*^)^*T*^, and **ε**_*i*_ = (*ε*_*i*11_, *ε*_*i*21_, *ε*_*i*31_, *ε*_*i*12_, *ε*_*i*22_, *ε*_*i*32_)^*T*^. Here, the elements **x**_*i*0_ = (*x*_*i*10_, *x*_*i*20_, *x*_*i*30_)^*T*^ of the covariance matrix **x**_*i*_ = (**x**_*i*0_, **x**_*i*1_, **x**_*i*2_) is a 3 × 1 vector of all ones. The elements **x**_*i*1_ = (*x*_*i*11_, *x*_*i*21_, *x*_*i*31_)^*T*^ of **x**_*i*_ are independently generated with an equal probability of being 0 or 1. The elements **x**_*i*2_ = (*x*_*i*12_, *x*_*i*22_, *x*_*i*32_)^*T*^ of **x**_*i*_ are generated from a multivariate normal distribution with a mean of 0.5 and a covariance matrix with diagonal entries of 1 and all off-diagonal entries of 0.1. The regression coefficients of the covariate matrix **x**_*i*_ for the *k*^th^ correlated trait are given by **α**_*k*_ = (*α*_0*k*_, *α*_1*k*_, *α*_2*k*_)^*T*^ = (0.01, 0.1, 0.1)^*T*^ and **α**_*k*_ = (*α*_0*k*_, *α*_1*k*_, *α*_2*k*_)^*T*^ = (−1.4, 0.1, 0.1)^*T*^, respectively, for continuous traits and binary traits for *k* = 1, 2.

For continuous traits, the error terms **ε**_*i*_ = (*ε*_*i*11_, *ε*_*i*21_, *ε*_*i*31_, *ε*_*i*12_, *ε*_*i*22_, *ε*_*i*32_)^*T*^ in equation (11) follow a multivariate normal distribution having a mean of zero, a within-in cluster correlation matrix (i.e., Cor(*ε*_*ijk*_, *ε*_*ij*′*k*_)) with diagonal entries of 1 and all off-diagonal entries of 0.2 and a subject-across-response correlation matrix (i.e., Cor(*ε*_*ijk*_, *ε*_*ij*′*k*′_)) with diagonal entries of 0.3 and all off-diagonal entries of 0.1. Similarly, binary traits **y**_*i*_ in equation (12) are generated with the same within-in cluster correlation matrix (i.e., Cor(*y*_*ijk*_, *y*_*ij*′*k*_)) and the same subject-across-response correlation matrix (i.e., Cor(*y*_*ijk*_, *y*_*ij*′*k*′_)) as the continuous traits **y**_*i*_ in equation (11). These correlated phenotypes are generated by the R package BinNor [53].

For type I error simulations, the regression coefficients of genetic variants, **β** = (**β**_1_^*T*^, **β**_2_^*T*^)^*T*^, in equations (11) and (12) are equal to zero under the null hypothesis. For power simulations, under the alternative hypothesis, we simulate that 35% of low variants with the MAF < 0.03 are causal. For each setting, either all causal SNPs have a positive effect, or 80% of causal SNPs are positive, and 20% of causal SNPs are negative. The regression coefficients of genetic variants, **β** = (**β**_1_^*T*^, **β**_2_^*T*^)^*T*^, are set by 0.095 × ∣log_10_(*m*_*l*_)∣ or 0.095 × log_10_(*m*_*l*_) corresponding to the risk or protective variant *l*, *l* = 1, 2, ⋯, *p* [51]. Under the assumption that the genetic effects on the two different phenotypes are heterogeneous (i.e., **β**_1_ ≠ **β**_2_), the genetic effects **β**_1_ for the first traits **y**_*i*1_ are set as described above, while the genetic effects **β**_2_ for the second traits **y**_*i*2_ are set by zero. On the other hand, under the assumption that the genetic effects on the two different phenotypes are homogeneous (i.e., **β**_1_ = **β**_2_), the genetic effects **β**_1_ and **β**_2_ for the first and second traits have the same settings as described above.

We simulate 1,400 nuclear families with 800 nuclear families from the European samples and 600 nuclear families from African-American samples. The marker-specific weight *w*_*l*_ for variant *l* is considered as the beta density function *w*_*l*_ = Beta(*m*_*l*_, *λ*_1_, *λ*_2_) with shape parameters *λ*_1_ > 0 and *λ*_2_ > 0 [51]. To study the effect of the marker-specific weight *w*_*l*_ of variant *l* on the phenotypes, we consider the unweighted marker-specific weight with *w*_*l*_ = Beta(*m*_*l*_, 1, 1) = 1 and the weighted marker-specific weight with *w*_*l*_ = Beta(*m*_*l*_, 1, 25) [51]. The empirical type I error rates based on fifty thousand replicates and the empirical power rates based on two thousand replicates are reported for all simulation results. The “exchangeable” and “unstructured” structures are considered for the working within-cluster and multivariate-response correlation matrices for the proposed methods, HoK, HeK, and BT, respectively.

## 4. Results

### 4.1. Empirical Type I Error Rates

Table 1 reports the results of a simulation comparison on empirical type I error rates when the phenotypes are considered to be continuous. Table 1 displays that the proposed methods, HoK, HoO, HeK, HeO, and BT, well control the empirical type I error rates regardless of the weight of the marker-specific weight. Similarly, the existing methods, mPK and mPB, have good performance on controlling the empirical type I error rates. Our simulation results show that the seven competing methods, HoK, HoO, HeK, HeO, BT, mPK, and mPB, reasonably control the empirical type I error rates for autosome analyses with continuous traits. The seven competing approaches display similar performance in terms of the empirical type I error rates for the X chromosome analyses with continuous traits (Appendix S3: Table S1).

Table 2 reports the empirical type I error rates based on the proposed methods, HoK, HeK, BT, HoO, and HeO, for the binary data. The two existing methods, mPK and mPB, aren't included for comparison. This reason is that implementing the two existing methods, mPK and mPB, via the R package Multi-SKAT [39], the MPMM (multiple phenotype mixed model) function in the R package PHENIX [54–56] is a necessary tool for this process. However, the MPMM function is suitable for the continuous phenotypes [56] or is suitable for the binary phenotypes with the condition that the number of cases is sufficiently large [39]. In other words, in some sense, the two existing methods, mPK and mPB, are limited to continuous phenotypes [39].

Table 2 shows that the proposed methods appropriately control the type I error rates when the marker-specific weight is considered for *w*_*l*_ = Beta(*m*_*l*_, 1, 1) or *w*_*l*_ = Beta(*m*_*l*_, 1, 25) for variant *l* for binary traits. On the other hand, the empirical type I error rates of the proposed methods for X chromosome analyses with binary traits are depicted in Table S2 in Appendix S3. These empirical type I error rates show similar results as that for autosome analyses.

In summary, our simulation results show that the proposed multivariate trait association methods, HoK, HoO, HeK, HeO, and BT, have reasonable control of type I error rates for continuous traits or binary traits whether the marker is X chromosomal or autosomal. On the other hand, the existing methods, mPK and mPB, yield well-controlled type I error rates for the autosome analyses or the X chromosome analyses with continuous traits (Table 1 or Table S1), regardless of the weight of the marker-specific weight.

### 4.2. Empirical Power

**Figure 1:**
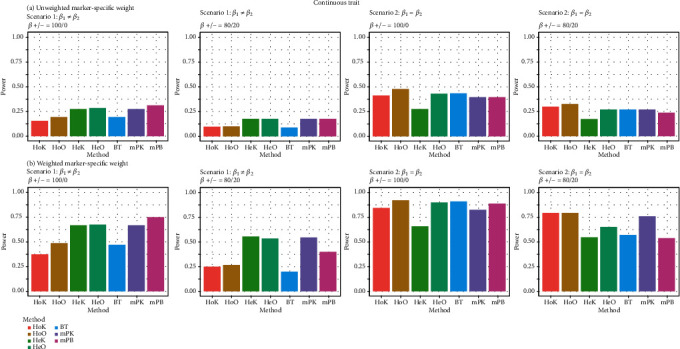
Power comparisons of the seven competing methods with continuous traits for each scenario at the nominal level of 0.001. (a) Unweighted marker-specific weight: *w*_*l*_ = Beta(*m*_*l*_, 1, 1) = 1. (b) Weighted marker-specific weight: *w*_*l*_ = Beta(*m*_*l*_, 1, 25).

Figure 1 exhibits the comparison results of the empirical power rates for the autosome analyses with continuous traits, when the working within-cluster and multivariate-response correlation matrices of the proposed methods, HoK, HeK, and BT, are considered to be exchangeable. As expected, the empirical power rates of the seven competing methods with a weighted marker-specific weight of *w*_*l*_ = Beta(*m*_*l*_, 1, 25) are higher than that with an unweighted marker-specific weight of *w*_*l*_ = Beta(*m*_*l*_, 1, 1) = 1. The heterogeneous kernel statistic (HeK) has slightly greater empirical power rates than other methods, when the genetic effects on the different phenotypes are heterogeneous (i.e., **β**_1_ ≠ **β**_2_), and causal SNPs have positive effects or negative effects on phenotypes. On the other hand, the existing method, mPB, has bigger empirical power rates, when the genetic effects on the different phenotypes are heterogeneous (i.e., **β**_1_ ≠ **β**_2_), and all causal SNPs have a positive association on phenotypes. Moreover, the empirical power rates of the homogeneous omnibus test (HoO) are larger than that of the other six competing methods, when the genetic effects on the different phenotypes are homogeneous (i.e., **β**_1_ = **β**_2_). Evidently, the seven competing methods have their respective advantages in identifying the association between genetic effects and multiple continuous traits for autosome analyses.

Similar empirical power rates are obtained from the working within-cluster and multivariate-response correlation matrices of the proposed methods, HoK, HeK, and BT, considered to be unstructured. Hence, these empirical power rates are not shown in order to save space. On the other hand, the seven competing approaches display a similar performance in testing for the X chromosome analyses with continuous traits (Appendix S3: Figure S1).

**Figure 2:**
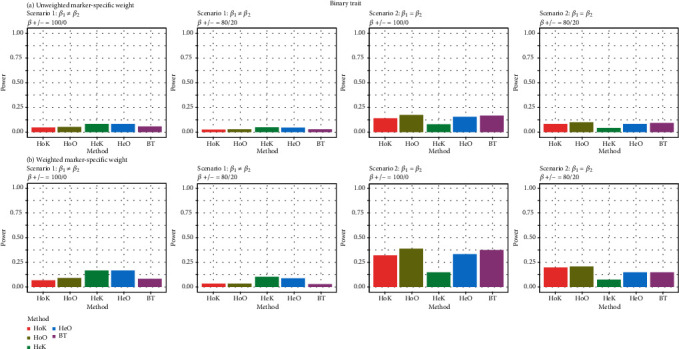
Power comparisons of the five competing methods with binary traits for each scenario at the nominal level of 0.001. (a) Unweighted marker-specific weight: *w*_*l*_ = Beta(*m*_*l*_, 1, 1) = 1. (b) Weighted marker-specific weight: *w*_*l*_ = Beta(*m*_*l*_, 1, 25).

Figure 2 exhibits the comparison results of empirical power rates for the autosome analyses with binary traits when the working within-cluster and multivariate-response correlation matrices of the proposed methods, HoK, HeK, and BT, are considered to be exchangeable. As a similar reason for investigating the empirical type I error rates with binary traits, the two existing methods, mPK and mPB, aren't included for power comparison.

Figure 2 shows that the heterogeneous kernel statistic (HeK) and the heterogeneous omnibus test (HeO) outperform over other methods in terms of the empirical power rates, when the genetic effects on the different phenotypes are heterogeneous (i.e., **β**_1_ ≠ **β**_2_). On the other hand, the empirical power rates of the homogeneous omnibus test (HoO) are bigger than that of the other competing methods, when the genetic effects on the different phenotypes are homogeneous (i.e., **β**_1_ = **β**_2_). As expected, in general, the heterogeneous kernel statistic (HeK) is more powerful than the homogeneous kernel statistic (HoK), when the genetic effects on the different phenotypes are heterogeneous (i.e., **β**_1_ ≠ **β**_2_). On the other hand, the homogeneous kernel statistic (HoK) is more powerful than the heterogeneous kernel statistic (HeK), when the genetic effects on the different phenotypes are homogeneous (i.e., **β**_1_ = **β**_2_). In a word, the proposed methods, HoK, HoO, HeK, HeO, and BT, have their respective merits in examining the association between genetic effects and multiple binary traits for autosome analyses.

Similarly, when the working within-cluster and multivariate-response correlation matrices of the proposed methods, HoK, HeK, and BT, are considered to be unstructured, the empirical power rates have similar results and thus they are omitted. On the other hand, the empirical power rates of the proposed methods for X chromosome analyses with binary traits are presented in Figure S2 in Appendix S3. These empirical power rates show similar results as that discussed in Figure 2.

In summary, the seven competing methods, HoK, HoO, HeK, HeO, BT, mPK, and mPB, have their respective merits in diagnosing whether genetic effects are associated with multiple continuous traits for autosome analyses or the X chromosome analyses. Similarly, the proposed methods, HoK, HoO, HeK, HeO, and BT, have their respective advantages in examining whether there are associations between genetic effects and multiple binary traits for autosome analyses or the X chromosome analyses.

To furthermore examine the performance of the proposed methods, additional simulation studies for continuous traits and binary traits are presented in Appendix S4 and Appendix S5 with higher correlations of phenotypes and higher dimensions of phenotypes considered, respectively. In general, these competing methods based on higher correlations of phenotypes or higher dimensions of phenotypes can provide a bigger empirical power rate for the analysis of continuous traits or binary traits. However, we note that these competing methods based on higher correlations of phenotypes or higher dimensions of phenotypes more easily have empirical type I error rate inflation at a smaller nominal level, especially for binary data analysis (Appendix S5: Tables S5-S6 and Appendix S6: Table S7), in comparison with these methods based on lower correlations of phenotypes or lower dimensions of phenotypes. A detailed discussion of these additional simulation results is given in Appendixes S4 and S5.

However, we note that the proposed methods have a high computational cost, especially for binary data. Under our simulation setting and framework, we carry out a single simulated data set by using a computer based on one CPU core at 2.1 GHz. The average computational times of the homogeneous and heterogeneous tests with a weighted marker-specific weight *w*_*l*_ = Beta(*m*_*l*_, 1, 25) under the alternative hypothesis for continuous data are 0.83 and 0.91 minutes, respectively, while that for binary data are 4.77 and 4.80 minutes, respectively. Therefore, in the current version, such a framework algorithm implementation is unsatisfactory for analyzing a large-scale high-dimensional data set in practice.

## 5. Conclusion

In this investigation, we develop a retrospective framework for identifying the pleiotropic effects of genetic variants on multivariate traits by using collapsing and kernel methods with pedigree- or population-structured data. The proposed framework, corresponding to the burden test, the kernel test, and the omnibus test, provides a sound basis for genetic association analyses for autosomes and the X chromosome. The proposed multivariate trait association methods based on the JGEE model can flexibly accommodate continuous phenotypes or binary phenotypes and further can adjust for covariates.

One critical advantage of the proposed methods is that the homogeneous kernel statistic (HoK), the heterogeneous kernel statistic (HeK), and the burden test (BT) retain all of the benefits of the retrospective tests proposed by Schaid et al. [43] who treated the genotype data as random variables by conditioning the phenotypes as constants. On the other hand, the homogeneous omnibus test (HoO) and the heterogeneous kernel statistic (HeO) keep the advantages of the Cauchy combination tests proposed by Liu and Xie [48] who showed that the Cauchy combination tests are robust to model misspecification and robustly protect the type I error rates [49].

Another important benefit of the proposed method is that the HoK test, the HeK test, and the BT test keep the benefits of the JGEE model that validly account for complex correlations between subjects within the cluster (within-cluster correlations) and between different phenotypes from the same subjects (multivariate-response correlations). Moreover, the proposed test statistics, HoK, HeK, and BT, based on the JGEE model can efficaciously account for covariate adjustment whether the phenotypes are continuous or binary.

Our simulation studies show that an unweighted marker-specific weight *w*_*l*_ = Beta(*m*_*l*_, 1, 1) = 1 and an exchangeable structure of the working within-cluster and multivariate-response correlations are recommended for the practical data analysis if the data cannot sufficiently provide valid information for estimating the structures of the working within-cluster and multivariate-response correlations before the start of the data analysis. Moreover, the homogeneous kernel statistic (HoK) is more robust than the heterogeneous statistic (HeK) in controlling the empirical type I errors, because the null distribution of the HeK statistic asymptotically follows a mixture chi-square distribution with a larger degree of freedom, in comparison with the null distribution of the HoK statistic. However, the HeK statistic is more powerful than the HoK statistic when the genetic effects on the different phenotypes are heterogeneous.

On the other hand, our simulation results show that for the autosome analyses or the X chromosome analyses with continuous traits, the seven competing methods, HoK, HoO, HeK, HeO, BT, mPK, and mPB, show good performance with well-controlled type I errors, while the seven competing methods have their respective merits for identifying the association between the genetic effects and multiple continuous traits. In addition, our simulation results show that for the autosome analyses or the X chromosome analyses with binary traits, the proposed methods, HoK, HoO, HeK, HeO, and BT, can control empirical type I errors with lower correlations of phenotypes or with lower dimensions of phenotypes (Table 2 and Table S2), while these proposed methods have their respective advantages for identifying the genetic variants associated with multiple binary traits. However, we observe that the proposed methods, HoK, HoO, HeK, HeO, and BT, with higher correlations of phenotypes or with higher dimensions of phenotypes, more easily have the infection of empirical type I errors at a smaller nominal level (Appendix S5: Tables S5-S6 and Appendix S6: Table S7), although these method under such situations have higher empirical power rates.

## 6. Limitation

The proposed multivariate trait association methods have their limitations. First, these proposed methods cannot simultaneously include the continuous traits and binary traits in analysis. Thus, future studies are needed to extend the idea of the proposed multivariate trait association methods for simultaneously considering continuous traits and binary traits in analysis. Second, the multivariate trait association methods, based on higher correlations of phenotypes or higher dimensions of phenotypes, easily suffer from the problem of the inflated type I errors, especially when the binary traits are considered (Appendix S5: Tables S5-S6 and Appendix S6: Table S7). Although the JGEE model provides an efficient algorithm for estimating the structure of the working within-cluster and multivariate-response correlations, a large-scale pedigree study always suffers from a more complex and high-dimensional structure of the within-cluster and multivariate-response correlations in pedigree database analysis. Hence, in the future, a more effective algorithm for estimating the complicated and high-dimensional (or higher correlational) structure of the working within-cluster and multivariate-response correlations is necessary to be proposed, especially when the analysis focuses on the binary traits. Third, in comparison with the null distribution of the homogeneous kernel statistic, the null distribution of the heterogeneous kernel statistic follows a larger degree of freedom test, which easily causes such a heterogeneous test to suffer from the problem of the type I error inflation. Therefore, overcoming the problem of the type I error inflation from the heterogeneous test is an essential part of the future work. Fourth, the proposed methods, which have a high computational cost especially for binary data, are inappropriate for analyzing large-scale high-dimensional data in practice. Thus, a more effective algorithm for reducing computational cost is needed to be proposed in further research. Moreover, the software of the proposed methods is computationally inconvenient and particularly inadequate for the mass GWAS data in practice. Therefore, the software of the proposed methods, which is convenient to be used, is a further work in the future. Fifth, our current work focuses mainly on the low- and common-frequency variants. Extension of the proposed methods to the rare variants deserves further works.

## Figures and Tables

**Table 1:** Empirical type I errors of the seven competing methods with continuous traits.

Marker-specific weight (*w*_*l*_)	Nominal level	Working correlation	Method						
			HoK^3^	HoO	HeK	HeO	BT	mPK^4^	mPB
Unweighted marker-specific weight^1^	0.05	U/U^2^	0.04876	0.04960	0.05036	0.05228	0.04914	0.04352	0.04692
		E/E	0.04866	0.04994	0.05016	0.05216	0.04914		
	0.01	U/U	0.00918	0.01012	0.01016	0.01030	0.01034	0.00854	0.01036
		E/E	0.00924	0.00994	0.01008	0.01022	0.01028		
	0.001	U/U	0.00078	0.00082	0.00086	0.00070	0.00084	0.00084	0.00088
		E/E	0.00080	0.00078	0.00084	0.00070	0.00082		
	0.0001	U/U	0.00008	0.00002	0.00006	0.00008	0.00008	0.00006	0.00014
		E/E	0.00006	0.00002	0.00006	0.00008	0.00008		
Weighted marker-specific weight	0.05	U/U	0.05030	0.04998	0.05158	0.05134	0.04696	0.04604	0.04536
		E/E	0.05054	0.05010	0.05176	0.05122	0.04714		
	0.01	U/U	0.00992	0.00942	0.01080	0.00972	0.00888	0.00978	0.01008
		E/E	0.00992	0.00944	0.01088	0.00978	0.00886		
	0.001	U/U	0.00078	0.00086	0.00126	0.00098	0.00082	0.00124	0.00134
		E/E	0.00076	0.00088	0.00122	0.00102	0.00080		
	0.0001	U/U	0.00006	0.00008	0.00006	0.00006	0.00010	0.00002	0.00010
		E/E	0.00006	0.00008	0.00008	0.00006	0.00010		

^1^The unweighted marker-specific weight is given by *w*_*l*_ = Beta(*m*_*l*_, 1, 1) = 1; the weighted marker-specific weight is given by *w*_*l*_ = Beta(*m*_*l*_, 1, 25). ^2^U/U represents the structures of the working within-cluster and multivariate-response correlation matrices considered by the unstructured structures; E/E represents the structures of the working within-cluster and multivariate-response correlation matrices considered by the exchangeable structures. ^3^HoK, HoO, HeK, HeO, and BT are our proposed methods. ^4^mPK and mPB are executed by the R package Multi-SKAT [39].

**Table 2:** Empirical type I errors of the five competing methods with binary traits.

Marker-specific weight (*w*_*l*_)	Nominal level	Working correlation	Method				
			HoK^3^	HoO	HeK	HeO	BT
Unweighted marker-specific weight^1^	0.05	U/U^2^	0.04944	0.05154	0.05086	0.05280	0.04952
		E/E	0.04930	0.05144	0.05068	0.05318	0.04946
	0.01	U/U	0.00974	0.00994	0.00982	0.01026	0.01000
		E/E	0.00974	0.00998	0.00984	0.01028	0.00998
	0.001	U/U	0.00068	0.00084	0.00100	0.00098	0.00106
		E/E	0.00066	0.00084	0.00102	0.00094	0.00104
	0.0001	U/U	0.00008	0.00002	0.00012	0.00010	0.00000
		E/E	0.00008	0.00002	0.00012	0.00010	0.00002
Weighted marker-specific weight	0.05	U/U	0.05170	0.04900	0.05256	0.04922	0.04576
		E/E	0.05168	0.04920	0.05232	0.04930	0.04556
	0.01	U/U	0.01028	0.00976	0.00996	0.00972	0.00886
		E/E	0.01024	0.00982	0.00986	0.00976	0.00884
	0.001	U/U	0.00110	0.00080	0.00096	0.00090	0.00088
		E/E	0.00112	0.00076	0.00096	0.00088	0.00090
	0.0001	U/U	0.00004	0.00008	0.00010	0.00012	0.00006
		E/E	0.00006	0.00008	0.00010	0.00012	0.00008

^1^The unweighted marker-specific weight is given by *w*_*l*_ = Beta(*m*_*l*_, 1, 1) = 1; the weighted marker-specific weight is given by *w*_*l*_ = Beta(*m*_*l*_, 1, 25). ^2^U/U represents the structures of the working within-cluster and multivariate-response correlation matrices considered by the unstructured structures; E/E represents the structures of the working within-cluster and multivariate-response correlation matrices considered by the exchangeable structures. ^3^HoK, HoO, HeK, HeO, and BT are our proposed methods.

## Data Availability

The data supporting the findings of this study are available within the article and its supplementary materials.

## Supplementary Materials

Supplementary Materials Appendix S1: the null distribution of the kernel statistic. Appendix S2: extension to the X chromosome. Appendix S3: simulation results based on the X chromosome. Appendix S4: additional simulation studies for continuous traits. Appendix S5: additional simulation studies for binary traits. Appendix S6: limitation
