# Supplementary material for: Associating Multivariate Traits with Genetic Variants Using Collapsing and Kernel Methods with Pedigree- or Population-Based Studies
Source: Comput Math Methods Med. 2021 Feb 9;2021:8812282. doi: 10.1155/2021/8812282 (PMC7889379; doi:10.1155/2021/8812282)
Supplement: Supplementary Materials — Appendix S1: the null distribution of the kernel statistic. Appendix S2: extension to the X chromosome. Appendix S3: simulation results based on the X chromosome. Appendix S4: additional simulation studies for continuous traits. Appendix S5: additional simulation studies for binary traits. Appendix S6: limitation [file 8812282.f1.pdf]

Associating Multivariate Traits with Genetic Variants Using Collapsing and Kernel Methods with Pedigree- or Population-Based Studies

Supplementary Materials

Li-Chu Chien*

Center for Fundamental Science, Kaohsiung Medical University, Kaohsiung, Taiwan

*To whom correspondence should be addressed.

Contact: lcchien@kmu.edu.tw

Contents

Appendix S1. The Null Distribution of the Kernel Statistic 2

The Null Distribution of κ_{Ho} 2

The Null Distribution of κ_{He} 3

Appendix S2. Extension to the X Chromosome 4

Kernel Statistic 5

Burden Test 7

Omnibus Test 7

Appendix S3. Simulation Results Based on the X Chromosome 10

Empirical Type I Error Rates 10

Empirical Power 13

Appendix S4. Additional Simulation Studies for Continuous Traits 18

4.1. Simulation Results with Higher Correlations of Phenotype Traits 18

4.2. Simulation Results with the Dimension of Phenotypes Given by K = 3 21

Appendix S5. Additional Simulation Studies for Binary Traits 26

5.1. Simulation Results with Slightly Higher Correlations of Phenotype Traits 26

5.2. Simulation Results with the Dimension of Phenotypes Given by K = 3 30

Appendix S6. Limitation 35

Appendix S1. The Null Distribution of the Kernel Statistic

The Null Distribution of κ_{Ho}

Assuming Hardy-Weinberg equilibrium, the expectation of the random variables for genotypes, under the null hypothesis of no association between genetic effects and traits, is $E_0(g_{ijl}) = 2m_l$ [1]. Let $\tilde{\boldsymbol{Z}}_{\text{Ho}} = (\tilde{Z}_1, \tilde{Z}_2, \cdots, \tilde{Z}_p)^T$ be a $p \times 1$ vector with elements $\tilde{Z}_l = w_l Z_l$ for $l = 1, 2, \cdots, p$. Based on the multivariate central limit theorem, $\tilde{\boldsymbol{Z}}_{\text{Ho}}$ under the null hypothesis has an approximately multivariate normal distribution with mean $E_0(\tilde{\boldsymbol{Z}}_{\text{Ho}})$ and covariance matrix $\text{Cov}_0(\tilde{\boldsymbol{Z}}_{\text{Ho}})$. The values of the null mean of $\tilde{\boldsymbol{Z}}_{\text{Ho}}$ are zero, $E_0(\tilde{Z}_l) = w_l 2m_l \sum_{k=1}^{K} \sum_{i=1}^{N} \boldsymbol{1}_i^T \hat{\Delta}_{ik} \hat{\boldsymbol{A}}_{ik} \boldsymbol{S}_{ik} = 0$, $l = 1, 2, \ldots, p$, where $\boldsymbol{1}_i$ is a $n_i \times 1$ vector including all elements of 1, when $\boldsymbol{\mu}_i$, $i = 1, 2, \cdots, N$, are correctly specified. That is because, the elements $\hat{\boldsymbol{\mu}}_i$, $\hat{\boldsymbol{V}}_i$, $\hat{\Delta}_i$ and $\hat{\boldsymbol{A}}_i$ are consistent estimates of $E_0(\boldsymbol{y}_i)$, $\text{Cov}_0(\boldsymbol{y}_i)$, Δ_i and $\boldsymbol{A}_i$ under the null hypothesis, when the number of pedigree *N* is large enough. The null covariance matrix of $\tilde{\boldsymbol{Z}}_{\text{Ho}}$ is

$$\begin{aligned}\text{Cov}_0(\tilde{Z}_l, \tilde{Z}_{l'}) &= w_l w_{l'} \sum_{i=1}^{N} \left[\left(\sum_{k=1}^{K} \boldsymbol{S}_{ik}^T \hat{\boldsymbol{A}}_{ik} \hat{\Delta}_{ik} \right) \text{Cov}_0(\boldsymbol{g}_{il}, \boldsymbol{g}_{il'}) \left(\sum_{k=1}^{K} \hat{\Delta}_{ik} \hat{\boldsymbol{A}}_{ik} \boldsymbol{S}_{ik} \right) \right] \\ &= 2C_{\text{Ho}} w_l w_{l'} H_{ll'} \sqrt{m_l(1-m_l) m_{l'}(1-m_{l'})}\end{aligned}$$

where $\text{Cov}_0(\boldsymbol{g}_{il}, \boldsymbol{g}_{il'}) = 2 \times \Omega_i \times \left\{ H_{ll'} \sqrt{m_l(1-m_l) m_{l'}(1-m_{l'})} \right\}$, $C_{\text{Ho}} = \sum_{i=1}^{N} \left[\left(\sum_{k=1}^{K} \boldsymbol{S}_{ik}^T \hat{\boldsymbol{A}}_{ik} \hat{\Delta}_{ik} \right) \Omega_i \left(\sum_{k=1}^{K} \hat{\Delta}_{ik} \hat{\boldsymbol{A}}_{ik} \boldsymbol{S}_{ik} \right) \right]$ and Ω_i is a $n_i \times n_i$ matrix of genetic correlations for all n_i individuals in the ith pedigree. Here the genetic correlations Ω_i have the same definition as that given by Schaid et al. [1]. Hence, the null distribution of κ_{Ho} asymptotically

follows a mixture chi-square distribution $\sum_{l=1}^{p} \lambda_l \chi_{l,1}^2$, where $\chi_{l,1}^2$ s are independent random variables following a chi-square distribution with one degree of freedom and $(\lambda_1, \lambda_2, \ldots, \lambda_p)$ are nonzero eigenvalues of the null covariate matrix of

$\mathrm{Cov}_0(\tilde{Z}_l, \tilde{Z}_{l'}) = 2C_{\mathrm{Ho}} w_l w_{l'} H_{ll'} \sqrt{m_l(1-m_l)m_{l'}(1-m_{l'})}$.

The Null Distribution of κ_{He}

Let $\tilde{\boldsymbol{Z}}_{\mathrm{He}} = (\tilde{Z}_{11}, \cdots, \tilde{Z}_{1K}, \cdots, \tilde{Z}_{p1}, \cdots, \tilde{Z}_{pK})^T$ be a $(p \times K) \times 1$ vector with elements $\tilde{Z}_{lk} = w_{lk} Z_{lk}$ for $l = 1, 2, \cdots, p, k = 1, 2, \cdots, K$. Processing the similar procedure for derivation of the null distribution of κ_{Ho}, the null distribution of $\tilde{\boldsymbol{Z}}_{\mathrm{He}}$ asymptotically follows a multivariate normal distribution having zero mean and covariance matrix $\mathrm{Cov}_0(\tilde{\boldsymbol{Z}}_{\mathrm{He}})$. The elements of $\mathrm{Cov}_0(\tilde{\boldsymbol{Z}}_{\mathrm{He}})$ are

$$\begin{aligned}\mathrm{Cov}_0(\tilde{Z}_{lk}, \tilde{Z}_{l'k'}) &= w_{lk} w_{l'k'} \sum_{i=1}^{N} \left[\boldsymbol{S}_{ik}^T \hat{\boldsymbol{A}}_{ik} \hat{\Delta}_{ik} \mathrm{Cov}_0(\boldsymbol{g}_{il}, \boldsymbol{g}_{il'}) \hat{\Delta}_{ik'} \hat{\boldsymbol{A}}_{ik'} \boldsymbol{S}_{ik'} \right] \\ &= 2C_{\mathrm{He}} w_{lk} w_{l'k'} H_{ll'} \sqrt{m_l(1-m_l)m_{l'}(1-m_{l'})}\end{aligned}$$

where $C_{\mathrm{He}} = \sum_{i=1}^{N} \left[\boldsymbol{S}_{ik}^T \hat{\boldsymbol{A}}_{ik} \hat{\Delta}_{ik} \Omega_i \hat{\Delta}_{ik'} \hat{\boldsymbol{A}}_{ik'} \boldsymbol{S}_{ik'} \right]$. Therefore, the null distribution of κ_{He} asymptotically follows a mixture chi-square distribution $\sum_{l=1}^{(p \times K)} \lambda_l \chi_{l,1}^2$, where $\chi_{l,1}^2$ s are independent random variables following a chi-square distribution with one degree of freedom and $(\lambda_1, \lambda_2, \ldots, \lambda_{(p \times K)})$ are nonzero eigenvalues of the null covariate matrix of

$\mathrm{Cov}_0(\tilde{Z}_{lk}, \tilde{Z}_{l'k'}) = 2C_{\mathrm{He}} w_{lk} w_{l'k'} H_{ll'} \sqrt{m_l(1-m_l)m_{l'}(1-m_{l'})}$.

Appendix S2. Extension to the X Chromosome

To extend our methods to the X chromosome, we follow the idea from Schaid et al. [1] to use d to describe the code for men carrying the minor allele. Precisely, men are coded as 0 or d (d = 1 or 2), while women are coded as 0, 1, or 2 (as for autosomes) [1]. Under the null hypothesis of no association between genotypes and phenotypes, the covariance matrix of the genotype codes for the X chromosome is given by

$$\mathrm{Cov}_0^{\mathrm{X}}(\boldsymbol{g}_{il}, \boldsymbol{g}_{il'}) = \left(\Omega_i^{\mathrm{X}} * \boldsymbol{\alpha}_i\right) \times \left\{H_{ll'}\sqrt{m_l(1-m_l)m_{l'}(1-m_{l'})}\right\}$$

which has the same interpretation and definition in Schaid et al. [1]. Here the notation $*$ represents the element-wise multiplication. The genetic correlation Ω_i^{X} for the X chromosome for all n_i individuals in the ith pedigree has the same definition in equation (2) in Schaid et al. [1]. Similarly, $\boldsymbol{\alpha}_i$ is a $n_i \times n_i$ matrix with the elements given by [1]

$$\boldsymbol{\alpha}_i = \begin{cases} 2 & \text{if female-}j\text{ and female-}j'\text{ pair,} \\ d^2 & \text{if male-}j\text{ and male-}j'\text{ pair,} \\ d\sqrt{2} & \text{if female-}j\text{ and male-}j'\text{ pair,} \end{cases}$$

which has the same interpretation and definition as that given by Schaid et al. [1].

When the genetic relationship between subjects j and j' in the ith pedigree is unknown, the elements of the genetic correlation Ω_i^{X} for the X chromosome can be estimated through genomic data [1, 2] and its estimate is given by [1]

$$\hat{\Omega}_i^{\mathrm{X}} = \begin{cases} \frac{1}{p}\sum_{l=1}^{p}\frac{(g_{ijl}-2m_l)(g_{ij'l}-2m_l)}{2m_l(1-m_l)} & \text{if female-}j\text{ and female-}j'\text{ pair,} \\ \frac{1}{p}\sum_{l=1}^{p}\frac{(g_{ijl}-m_l)(g_{ij'l}-m_l)}{m_l(1-m_l)} & \text{if male-}j\text{ and male-}j'\text{ pair,} \\ \frac{1}{p}\sum_{l=1}^{p}\frac{(g_{ijl}-m_l)(g_{ij'l}-2m_l)}{\sqrt{2}m_l(1-m_l)} & \text{if male-}j\text{ and female-}j'\text{ pair.} \end{cases}$$

Based on the null covariance matrix of the genotype codes for the X chromosome, $\mathrm{Cov}_0^{\mathrm{X}}(\boldsymbol{g}_{il}, \boldsymbol{g}_{il'}) = \left(\Omega_i^{\mathrm{X}} * \boldsymbol{\alpha}_i\right) \times \left\{H_{ll'}\sqrt{m_l(1-m_l)m_{l'}(1-m_{l'})}\right\}$, we extend the homogeneous kernel statistic (κ_{Ho}), the heterogeneous kernel statistic (κ_{He}) and the burden test (BT) to the X chromosome.

Kernel Statistic

<u>Homogeneous Kernel Statistic</u>

Under the assumption that the genetic effects on the *K* different phenotypes are homogeneous (i.e., $\boldsymbol{\beta}_1 = \boldsymbol{\beta}_2 = \cdots = \boldsymbol{\beta}_K$), the null covariance matrix of $\boldsymbol{Z}_{\mathrm{Ho}} = (\tilde{Z}_1, \tilde{Z}_2, \cdots, \tilde{Z}_p)^T$ for the X chromosome is given by

$$\begin{aligned} \mathrm{Cov}_0^{\mathrm{X}}(\tilde{Z}_l, \tilde{Z}_{l'}) &= w_l w_{l'} \sum_{i=1}^{N}\left[\left(\sum_{k=1}^{K}\boldsymbol{S}_{ik}^T\hat{\boldsymbol{A}}_{ik}\hat{\Delta}_{ik}\right)\mathrm{Cov}_0^{\mathrm{X}}(\boldsymbol{g}_{il}, \boldsymbol{g}_{il'})\left(\sum_{k=1}^{K}\hat{\Delta}_{ik}\hat{\boldsymbol{A}}_{ik}\boldsymbol{S}_{ik}\right)\right] \\ &= C_{\mathrm{Ho}}^{\mathrm{X}} w_l w_{l'} H_{ll'}\sqrt{m_l(1-m_l)m_{l'}(1-m_{l'})} \end{aligned}$$

where $C_{\mathrm{Ho}}^{\mathrm{X}} = \sum_{i=1}^{N}\left[\left(\sum_{k=1}^{K}\boldsymbol{S}_{ik}^T\hat{\boldsymbol{A}}_{ik}\hat{\Delta}_{ik}\right)\left(\Omega_i^{\mathrm{X}} * \boldsymbol{\alpha}_i\right)\left(\sum_{k=1}^{K}\hat{\Delta}_{ik}\hat{\boldsymbol{A}}_{ik}\boldsymbol{S}_{ik}\right)\right]$. Therefore, the null distribution of $\kappa_{\mathrm{Ho}} = \sum_{l=1}^{p}\tilde{Z}_l^2$ for the X chromosome asymptotically follows a mixture chi-square distribution

$\sum_{l=1}^{p}\lambda_l\chi_{l,1}^2$, where $\chi_{l,1}^2$s are independent random variables following a chi-square distribution with one degree of freedom and $(\lambda_1,\lambda_2,\ldots,\lambda_p)$ are nonzero eigenvalues of the null covariate matrix of $\text{Cov}_0^{\text{X}}(\tilde{Z}_l,\tilde{Z}_{l'})=C_{\text{Ho}}^{\text{X}}w_l w_{l'}H_{ll'}\sqrt{m_l(1-m_l)m_{l'}(1-m_{l'})}$.

<u>Heterogeneous Kernel Statistic</u>

Under the assumption that the genetic effects on the *K* different phenotypes are heterogeneous (i.e., $\boldsymbol{\beta}_1\neq\boldsymbol{\beta}_2\neq\cdots\neq\boldsymbol{\beta}_K$), the null covariance matrix of $\boldsymbol{Z}_{\text{He}}=(\tilde{Z}_{11},\cdots,\tilde{Z}_{1K},\cdots,\tilde{Z}_{p1},\cdots,\tilde{Z}_{pK})^T$ for the X chromosome is given by

$$\begin{aligned}\text{Cov}_0^{\text{X}}(\tilde{Z}_{lk},\tilde{Z}_{l'k'})&=w_{lk}w_{l'k'}\sum_{i=1}^{N}\left[\boldsymbol{S}_{ik}^T\hat{\boldsymbol{A}}_{ik}\hat{\Delta}_{ik}\text{Cov}_0(\boldsymbol{g}_{il},\boldsymbol{g}_{il'})\hat{\Delta}_{ik'}\hat{\boldsymbol{A}}_{ik'}\boldsymbol{S}_{ik'}\right]\\&=C_{\text{He}}^{\text{X}}w_{lk}w_{l'k'}H_{ll'}\sqrt{m_l(1-m_l)m_{l'}(1-m_{l'})}\end{aligned}$$

where $C_{\text{He}}^{\text{X}}=\sum_{i=1}^{N}\left[\boldsymbol{S}_{ik}^T\hat{\boldsymbol{A}}_{ik}\hat{\Delta}_{ik}\left(\Omega_i^{\text{X}}*\boldsymbol{\alpha}_i\right)\hat{\Delta}_{ik'}\hat{\boldsymbol{A}}_{ik'}\boldsymbol{S}_{ik'}\right]$. Therefore, the null distribution of κ_{He} for the X chromosome asymptotically follows a mixture chi-square distribution $\sum_{l=1}^{(p\times K)}\lambda_l\chi_{l,1}^2$, where $\chi_{l,1}^2$s are independent random variables following a chi-square distribution with one degree of freedom and $(\lambda_1,\lambda_2,\ldots,\lambda_{(p\times K)})$ are nonzero eigenvalues of the null covariate matrix of $\text{Cov}_0^{\text{X}}(\tilde{Z}_{lk},\tilde{Z}_{l'k'})=C_{\text{He}}^{\text{X}}w_{lk}w_{l'k'}H_{ll'}\sqrt{m_l(1-m_l)m_{l'}(1-m_{l'})}$. Theoretical *p*-values of κ_{Ho} and κ_{He} for the X chromosome are approximately calculated by Kuonen's saddlepoint method [3] and obtained by the R package *pchisqsum*.

Burden Test

Let $\tilde{\boldsymbol{g}}_i^T = \sum_{l=1}^{p} w_l \boldsymbol{g}_{il}^T$ be a weighted average of genotype scores for the ith pedigree. On the basis of the HoK test (κ_{Ho}) and the HeK test (κ_{He}) for the X chromosome having the same marker-specific weight of the lth variant for each trait k (i.e., $w_l = w_{lk}, k = 1, 2, \cdots, K$), we propose the burden test (BT) for the X chromosome as follows:

$$\text{BT} = \frac{\left[\sum_{i=1}^{N}\left(\sum_{k=1}^{K} \boldsymbol{S}_{ik}^T \hat{\boldsymbol{A}}_{ik} \hat{\Delta}_{ik}\right)\tilde{\boldsymbol{g}}_i\right]^2}{\sum_{i=1}^{N}\left[\left(\sum_{k=1}^{K} \boldsymbol{S}_{ik}^T \hat{\boldsymbol{A}}_{ik} \hat{\Delta}_{ik}\right)\text{Cov}_0^{\text{X}}(\tilde{\boldsymbol{g}}_i)\left(\sum_{k=1}^{K} \hat{\Delta}_{ik} \hat{\boldsymbol{A}}_{ik} \boldsymbol{S}_{ik}\right)\right]}$$

where the null covariance matrix of $\tilde{\boldsymbol{g}}_i$ for the X chromosome is given by

$$\begin{aligned}\text{Cov}_0^{\text{X}}(\tilde{\boldsymbol{g}}_i) &= \text{Cov}_0^{\text{X}}\left(\sum_{l=1}^{p} w_l \boldsymbol{g}_{il}\right) = \sum_{l=1}^{p} w_l^2 \text{Cov}_0^{\text{X}}(\boldsymbol{g}_{il}, \boldsymbol{g}_{il}) + 2\sum_{l=1}^{p}\sum_{l'=l+1}^{p} w_l w_{l'} \text{Cov}_0^{\text{X}}(\boldsymbol{g}_{il}, \boldsymbol{g}_{il'}) \\ &= \left(\Omega_i^{\text{X}} * \boldsymbol{\alpha}_i\right)\sum_{l=1}^{p}\sum_{l'=1}^{p} w_l w_{l'} H_{ll'} \sqrt{m_l(1-m_l)m_{l'}(1-m_{l'})}\end{aligned}.$$

Then,

$$\text{BT} = \frac{\left[\sum_{i=1}^{N}\left(\sum_{k=1}^{K} \boldsymbol{S}_{ik}^T \hat{\boldsymbol{A}}_{ik} \hat{\Delta}_{ik}\right)\tilde{\boldsymbol{g}}_i\right]^2}{\sum_{l=1}^{p}\sum_{l'=1}^{p} w_l w_{l'} H_{ll'} \sqrt{m_l(1-m_l)m_{l'}(1-m_{l'})}C_{\text{Ho}}^{\text{X}}}.$$

The null distribution of BT for the X chromosome asymptotically follows a chi-square distribution with one degree of freedom.

Omnibus Test

Let p_{Ho}^{X}, p_{He}^{X} and p_{BT}^{X} denote the *p*-values obtained by the HoK, HeK and BT statistics from the X chromosome. Based on the idea of the *p*-value combination method through the Cauchy distribution [4-6], we propose the homogeneous omnibus test (HoO) and heterogeneous omnibus test (HeO) for the X chromosome.

<u>Homogeneous Omnibus Test</u>

Combining the p_{Ho}^{X} with the p_{BT}^{X}, we construct the homogeneous omnibus test (HoO) for the X chromosome as follows:

$$O_{\text{Ho}} = -\frac{1}{2}\left[F_C^{-1}(p_{\text{Ho}}^{\text{X}}) + F_C^{-1}(p_{\text{BT}}^{\text{X}})\right]$$

where F_C^{-1} stands for the inverse cumulative distribution function of the standard Cauchy distribution.

<u>Heterogeneous Omnibus Test</u>

Combining the p_{He}^{X} with the p_{BT}^{X}, we construct the heterogeneous omnibus test (HeO) for the X chromosome as follows:

$$O_{\text{He}} = -\frac{1}{2}\left[F_C^{-1}(p_{\text{He}}^{\text{X}}) + F_C^{-1}(p_{\text{BT}}^{\text{X}})\right].$$

The null distributions of the O_{Ho} test and the O_{He} test for the X chromosome asymptotically follow a standard Cauchy distribution [4-6]. The *p*-values of the O_{Ho} test and the O_{He} test for the

X chromosome can be calculated by the R package *RNOmni* [7].

Appendix S3. Simulation Results Based on the X Chromosome

Following the same simulation set-up as those described in simulation studies in the text, the proposed methods, HoK, HeK, BT, HoO and HeO, are applied to the X chromosome with the genotype scores of men coded as 0 or 1. The empirical type I error rates based on fifty thousand replicates and the empirical power rates based on two thousand replicates are reported for all simulation results.

Empirical Type I Error Rates

Table S1 shows the empirical type I error rates of the seven competing methods for X chromosome analyses with continuous traits. Table S1 displays that the proposed methods, HoK, HoO, HeK, HeO and BT, appropriately control the empirical type I error rates whether the marker-specific weight is considered for $w_l = Beta(m_l, 1, 1) = 1$ or $w_l = Beta(m_l, 1, 25)$ for variant *l*. Similarly, the existing methods, mPK and mPB, yield well-controlled type I error rates.

In brief, the seven competing methods, HoK, HoO, HeK, HeO, BT, mPK and mPB, show good type I error performance for X chromosome analyses with continuous traits.

Table S1: Empirical type I errors of the seven competing methods with continuous traits based on the X chromosome.

Marker-specific weight (w_l)	Nominal level	Working correlation	Method						
			HoK[3]	HoO	HeK	HeO	BT	mPK[4]	mPB
Unweighted marker-specific weight[1]	0.05	U/U[2]	0.04506	0.04728	0.04660	0.04842	0.04694	0.04264	0.04494
		E/E	0.04542	0.04720	0.04690	0.04862	0.04692		
	0.01	U/U	0.00774	0.00808	0.00890	0.00850	0.00870	0.00792	0.00910
		E/E	0.00800	0.00824	0.00878	0.00862	0.00876		
	0.001	U/U	0.00062	0.00066	0.00060	0.00068	0.00052	0.00088	0.00088
		E/E	0.00064	0.00066	0.00060	0.00068	0.00056		
	0.0001	U/U	0.00008	0.00006	0.00002	0.00004	0.00006	0.00004	0.00012
		E/E	0.00008	0.00006	0.00002	0.00004	0.00006		
Weighted marker-specific weight	0.05	U/U	0.04784	0.04912	0.04956	0.04942	0.04716	0.04636	0.04530
		E/E	0.04866	0.04924	0.04998	0.04976	0.04726		
	0.01	U/U	0.00922	0.00906	0.01000	0.00932	0.00848	0.00958	0.00988
		E/E	0.00938	0.00912	0.01020	0.00942	0.00852		
	0.001	U/U	0.00078	0.00072	0.00106	0.00092	0.00088	0.00124	0.00132
		E/E	0.00078	0.00074	0.00118	0.00094	0.00086		
	0.0001	U/U	0.00006	0.00008	0.00006	0.00008	0.00010	0.00010	0.00008
		E/E	0.00006	0.00008	0.00006	0.00008	0.00010		

[1]The unweighted marker-specific weight is given by $w_l = Beta(m_l,1,1) = 1$; the weighted marker-specific weight is given by $w_l = Beta(m_l,1,25)$.

[2]U/U represents the structures of the working within-cluster and multivariate-response correlation matrices considered by the unstructured structures; E/E represents the structures of the working within-cluster and multivariate-response correlation matrices considered by the exchangeable structures.

[3]HoK, HoO, HeK, HeO and BT are our proposed methods.

[4]mPK and mPB are executed by the R package *MultiSKAT* [8].

Table S2 shows the empirical type I error rates of the proposed methods, HoK, HoO, HeK, HeO and BT, for X chromosome analyses with binary traits. Table S2 exhibits that these proposed methods reasonably control empirical type I error rates, when the marker-specific weight is considered for $w_l = Beta(m_l,1,1)$ or $w_l = Beta(m_l,1,25)$ for binary traits based on X

chromosome analyses.

Table S2: Empirical type I errors of the five competing methods with binary traits based on the X chromosome.

Marker-specific weight (w_l)	Nominal level	Working correlation	Method				
			HoK[3]	HoO	HeK	HeO	BT
Unweighted marker-specific weight[1]	0.05	U/U[2]	0.04642	0.04922	0.04770	0.05102	0.04830
		E/E	0.04662	0.04978	0.04798	0.05122	0.04862
	0.01	U/U	0.00872	0.00920	0.00930	0.00972	0.00974
		E/E	0.00890	0.00926	0.00950	0.00982	0.00988
	0.001	U/U	0.00066	0.00070	0.00096	0.00086	0.00066
		E/E	0.00068	0.00068	0.00092	0.00086	0.00068
	0.0001	U/U	0.00004	0.00002	0.00012	0.00004	0.00004
		E/E	0.00006	0.00002	0.00012	0.00006	0.00004
Weighted marker-specific weight	0.05	U/U	0.04908	0.04794	0.04936	0.04706	0.04516
		E/E	0.04914	0.04822	0.04954	0.04756	0.04498
	0.01	U/U	0.00954	0.00956	0.00960	0.00930	0.00860
		E/E	0.00992	0.00970	0.00976	0.00938	0.00866
	0.001	U/U	0.00090	0.00076	0.00092	0.00086	0.00084
		E/E	0.00100	0.00084	0.00094	0.00088	0.00088
	0.0001	U/U	0.00004	0.00008	0.00010	0.00014	0.00008
		E/E	0.00004	0.00008	0.00012	0.00014	0.00008

[1]The unweighted marker-specific weight is given by $w_l = Beta(m_l, 1, 1) = 1$; the weighted marker-specific weight is given by $w_l = Beta(m_l, 1, 25)$.

[2]U/U represents the structures of the working within-cluster and multivariate-response correlation matrices considered by the unstructured structures; E/E represents the structures of the working within-cluster and multivariate-response correlation matrices considered by the exchangeable structures.

[3]HoK, HoO, HeK, HeO and BT are our proposed methods.

In summary, these competing methods, HoK, HoO, HeK, HeO, BT, mPK and mPB, have good performance in controlling the type I error rates for X chromosome analyses with

continuous traits, regardless of the weight of the marker-specific weight. Moreover, the proposed methods, HoK, HoO, HeK, HeO and BT, are suitable for X chromosome analyses with binary traits.

Empirical Power

Figure S1 exhibits the comparison results of empirical power rates for X chromosome analyses with continuous traits, when the working within-cluster and multivariate-response correlation matrices of the proposed methods, HoK, HeK and BT, are considered to be exchangeable. As expected, the empirical power rates of the seven competing methods with a weighted marker-specific weight of $w_l = Beta(m_l, 1, 25)$ are bigger than that with an unweighted marker-specific weight of $w_l = Beta(m_l, 1, 1) = 1$. The empirical power rates of the heterogeneous kernel statistic (HeK) are slightly higher than that of the other methods, when the genetic effects on the different phenotypes are heterogeneous (i.e., $\boldsymbol{\beta}_1 \neq \boldsymbol{\beta}_2$) and causal SNPs have positive effects or negative effects on phenotypes. On the other hand, the empirical power rates of the existing method, mPB, are larger than that of the other methods, when the genetic effects on the different phenotypes are heterogeneous (i.e., $\boldsymbol{\beta}_1 \neq \boldsymbol{\beta}_2$) and all causal SNPs have a positive association on phenotypes.

Moreover, the empirical power rates of the homogeneous omnibus test (HoO) are greater

than that of the other six competing methods, when the genetic effects on the different phenotypes are homogeneous (i.e., $\boldsymbol{\beta}_1 = \boldsymbol{\beta}_2$). In addition, similar simulation results of the empirical power rates are obtained (results not shown), when the working within-cluster and multivariate-response correlation matrices of the proposed methods, HoK, HeK and BT, are considered to be unstructured. Obviously, the seven competing methods, HoK, HoO, HeK, HeO, BT, mPK and mPB, have their respective advantages in detecting the association between genetic effects and multiple continuous traits for X chromosome analyses.

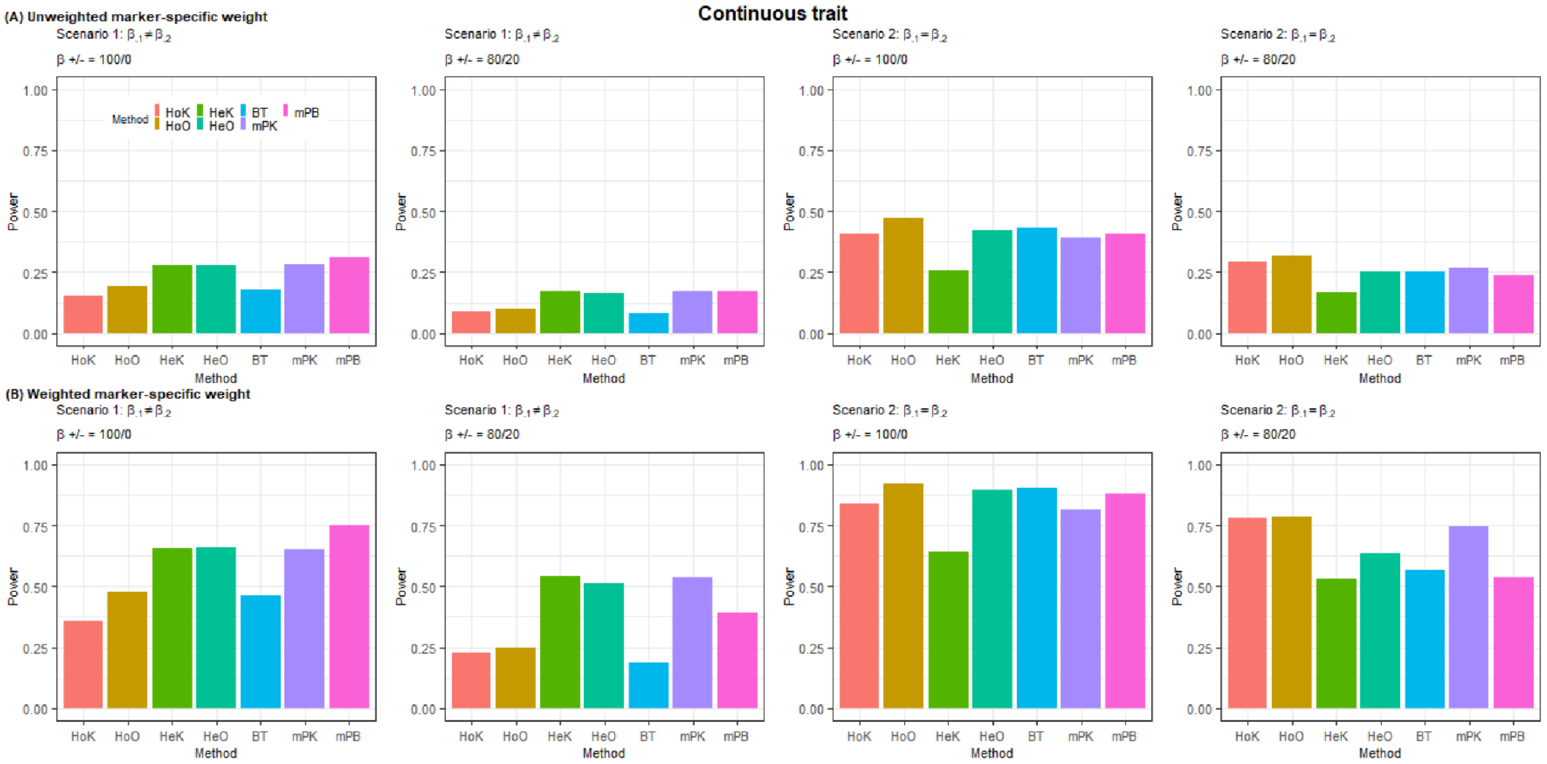

Figure S1: Power comparisons of the seven competing methods with continuous traits based on the X chromosome analyses for each scenario at the nominal level of 0.001. (A) Unweighted marker-specific weight: $w_l = Beta(m_l, 1, 1) = 1$. (B) Weighted marker-specific weight: $w_l = Beta(m_l, 1, 25)$.

Figure S2 exhibits the comparison results of empirical power rates for X chromosome analyses with binary traits when the working within-cluster and multivariate-response correlation matrices of the proposed methods, HoK, HeK and BT, are considered to be exchangeable. As expected, the empirical power rates of the heterogeneous kernel statistic (HeK) and the

heterogeneous omnibus test (HeO) are higher than that of the homogeneous kernel statistic (HoK) and the homogeneous omnibus test (HoO), when the genetic effects on the different phenotypes are heterogeneous (i.e., $\boldsymbol{\beta}_1 \neq \boldsymbol{\beta}_2$). On the other hand, the empirical power rates of the homogeneous omnibus test (HoO) are bigger than that of the other competing methods, when the genetic effects on the different phenotypes are homogeneous (i.e., $\boldsymbol{\beta}_1 = \boldsymbol{\beta}_2$). Clearly, the proposed methods, HoK, HoO, HeK, HeO and BT, have their respective benefits in identifying the association between genetic effects and multiple binary traits for X chromosome analyses.

In summary, the seven competing methods, HoK, HoO, HeK, HeO, BT, mPK and mPB, have their respective advantages in examining whether genetic effects are associated with multiple continuous traits for X chromosome analyses. On the other hand, the proposed methods, HoK, HoO, HeK, HeO and BT, have their respective merits in terms of the empirical power rates for binary traits for X chromosome analyses.

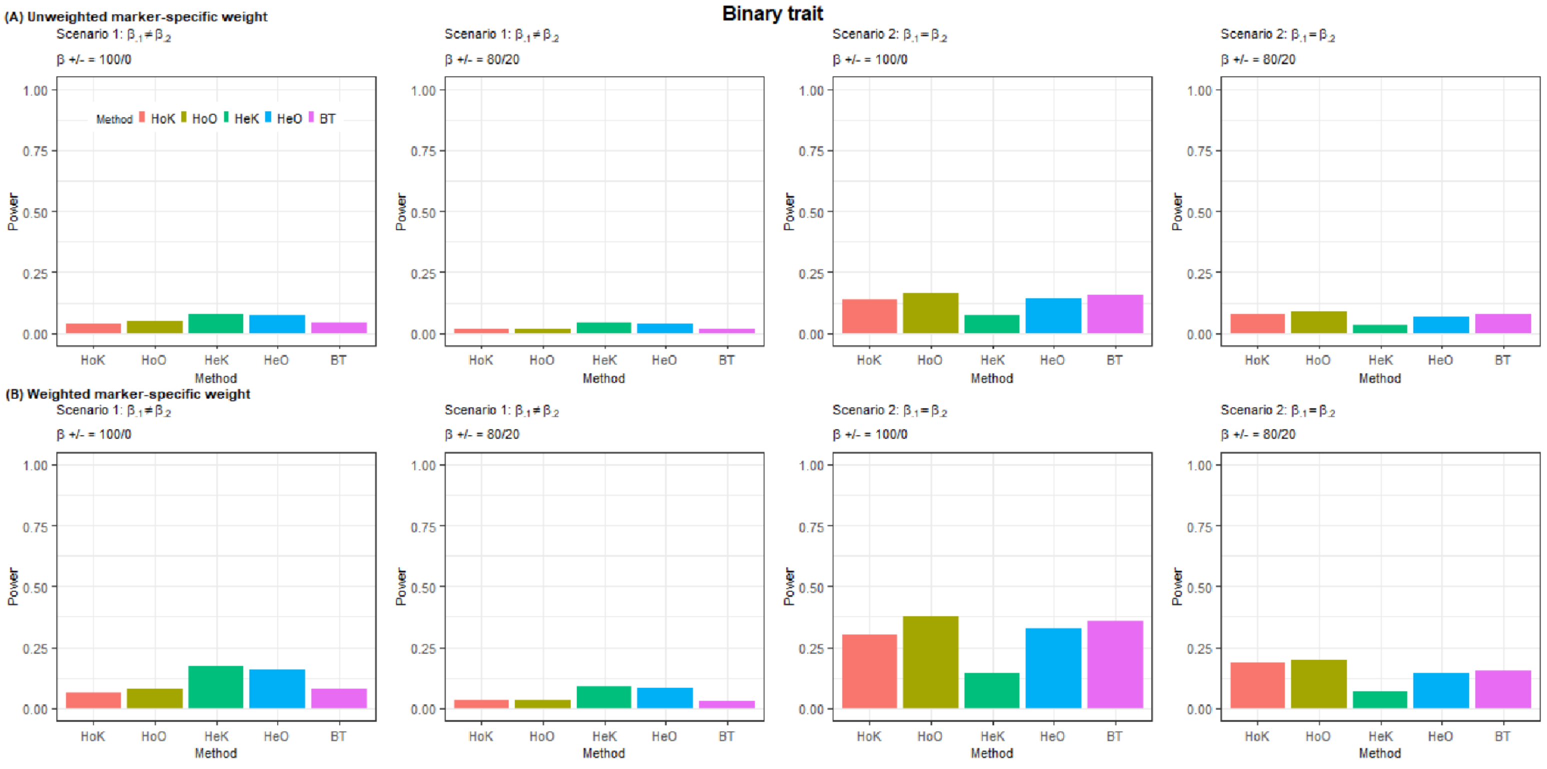

Figure S2: Power comparisons of the five competing methods with binary traits based on the X chromosome analyses for each scenario at the nominal level of 0.001. (A) Unweighted marker-specific weight: $w_l = Beta(m_l,1,1) = 1$. (B) Weighted marker-specific weight: $w_l = Beta(m_l,1,25)$.

Appendix S4. Additional Simulation Studies for Continuous Traits

In this section we present addition simlation results to futher examine the performance of the proposed methods with continuous traits.

4.1. Simulation Results with Higher Correlations of Phenotype Traits

Following the same simulation set-up as those described in simulation studies in the text, we consider the higher correlational relationships for continuous traits. Preciously, the error terms $\boldsymbol{\varepsilon}_i = (\varepsilon_{i11}, \varepsilon_{i21}, \varepsilon_{i31}, \varepsilon_{i12}, \varepsilon_{i22}, \varepsilon_{i32})^T$ in equation (11) are assumed to be from a multivariate normal distribution having a mean of zero, a within-in cluster correlation matrix (i.e., $\mathrm{Cor}(\varepsilon_{ijk}, \varepsilon_{ij'k})$) with diagonal entries of 1 and all off-diagonal entries of 0.7 and a subject-across-response correlation matrix (i.e., $\mathrm{Cor}(\varepsilon_{ijk}, \varepsilon_{ij'k'})$) with diagonal entries of 0.3 and all off-diagonal entries of 0.2. The empirical type I error rates based on fifty thousand replicates and the empirical power rates based on two thousand replicates are reported for all simulation results.

Empirical Type I Error Rates

Table S3 shows the empirical type I error rates of the seven competing methods, HoK, HoO, HeK, HeO, BT, mPK and mPB, with continuous traits based on higher correlations between phenotypes. Table S3 shows that the seven competing methods maintain reasonably empirical type I error rates when the marker-specific weight is considered for $w_l = Beta(m_l, 1, 1) = 1$ or

$w_l = Beta(m_l, 1, 25)$ for continuous traits.

In short, the seven competing methods can adequately control type I errors when continuous traits have higher correlations among one another.

Table S3: Empirical type I errors of the seven competing methods with continuous traits based on higher correlations between phenotypes.

Marker-specific weight (w_l)	Nominal level	Working correlation	Method						
			HoK[3]	HoO	HeK	HeO	BT	mPK[4]	mPB
Unweighted marker-specific weight[1]	0.05	U/U[2]	0.04376	0.04518	0.04420	0.04598	0.04672	0.04352	0.04692
		E/E	0.04426	0.04554	0.04424	0.04580	0.04672		
	0.01	U/U	0.00858	0.00880	0.00864	0.00880	0.00894	0.00854	0.01036
		E/E	0.00824	0.00878	0.00882	0.00890	0.00882		
	0.001	U/U	0.00092	0.00078	0.00060	0.00070	0.00076	0.00084	0.00088
		E/E	0.00086	0.00076	0.00058	0.00072	0.00078		
	0.0001	U/U	0.00002	0.00008	0.00002	0.00008	0.00006	0.00006	0.00014
		E/E	0.00002	0.00004	0.00004	0.00006	0.00006		
Weighted marker-specific weight	0.05	U/U	0.04798	0.04658	0.04908	0.04660	0.04264	0.04604	0.04536
		E/E	0.04806	0.04650	0.04870	0.04658	0.04284		
	0.01	U/U	0.00952	0.00884	0.00920	0.00866	0.00772	0.00978	0.01008
		E/E	0.00952	0.00856	0.00918	0.00856	0.00776		
	0.001	U/U	0.00082	0.00076	0.00100	0.00084	0.00086	0.00124	0.00134
		E/E	0.00080	0.00082	0.00100	0.00080	0.00078		
	0.0001	U/U	0.00006	0.00004	0.00006	0.00002	0.00008	0.00002	0.00010
		E/E	0.00006	0.00004	0.00010	0.00002	0.00006		

[1]The unweighted marker-specific weight is given by $w_l = Beta(m_l, 1, 1) = 1$; the weighted marker-specific weight is given by $w_l = Beta(m_l, 1, 25)$.

[2]U/U represents the structures of the working within-cluster and multivariate-response correlation matrices considered by the unstructured structures; E/E represents the structures of the working within-cluster and multivariate-response correlation matrices considered by the exchangeable structures.

[3]HoK, HoO, HeK, HeO and BT are our proposed methods.

[4]mPK and mPB are executed by the R package *MultiSKAT* [8].

Empirical Power

Figure S3 shows the empirical power rates of the seven competing methods, HoK, HoO, HeK, HeO, BT, mPK and mPB, with continuous traits based on higher correlations between phenotypes, when the working within-cluster and multivariate-response correlation matrices of the proposed methods, HoK, HeK and BT, are considered to be exchangeable. In comparison with the empirical power rates presented in Figure 1 and Figure S3, the empirical power rates based on the higher correlations of phenotypes in Figure S3 are larger than that based on the lower correlations of phenotypes in Figure 1. Moreover, the empirical power rates displayed in Figure 1 and Figure S3 have similar patterns, because both of them have similar correlation structures of phenotypes.

In summary, our simulation results show that, in general, the empirical power rates based on the higher correlations of phenotypes are bigger than that based on the lower correlations of phenotypes, when continuous traits are considered.

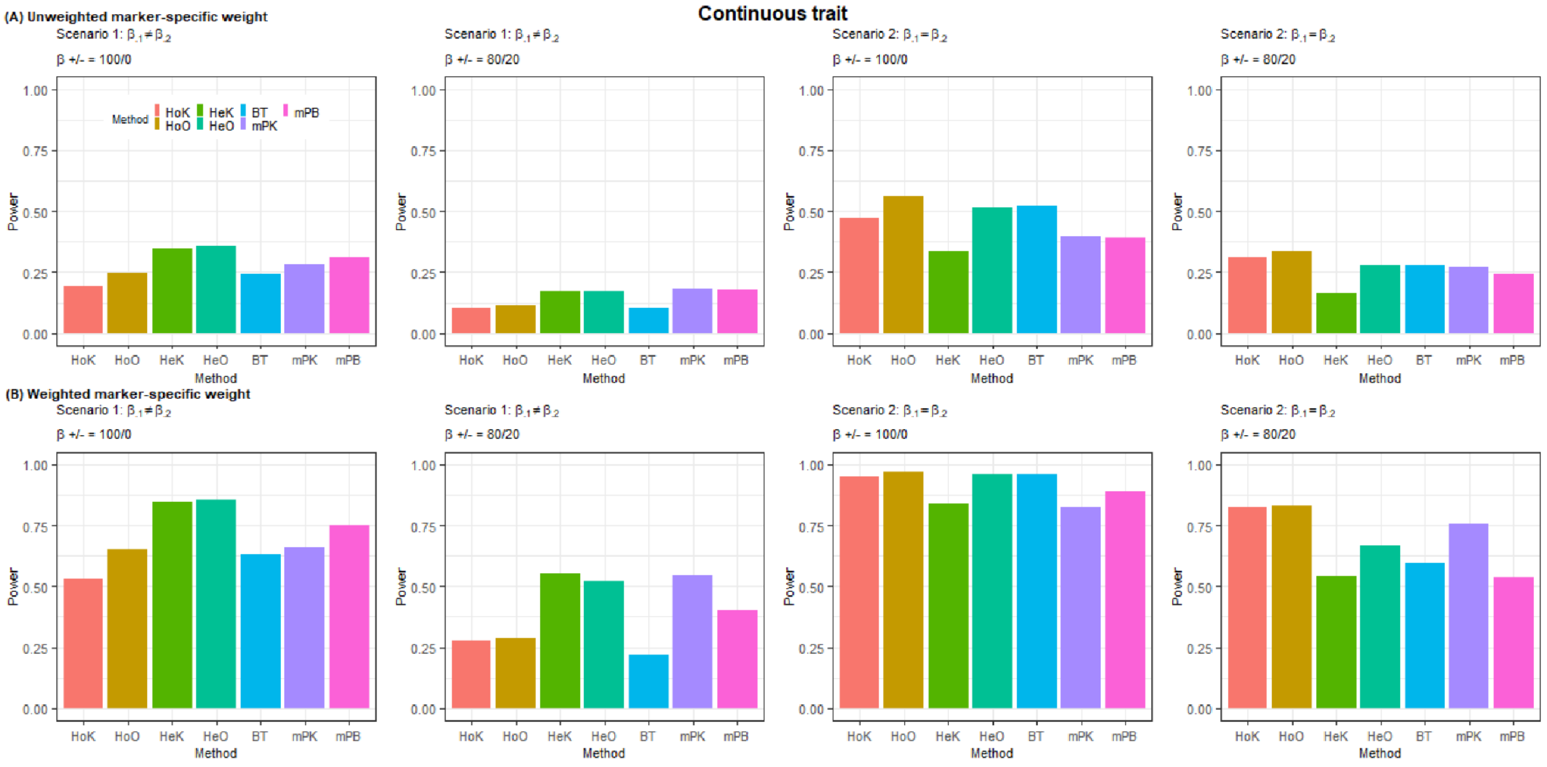

Figure S3: Power comparisons of the seven competing methods with continuous traits based on higher correlations between phenotypes for each scenario at the nominal level of 0.001. (A) Unweighted marker-specific weight: $w_l = Beta(m_l,1,1) = 1$. (B) Weighted marker-specific weight: $w_l = Beta(m_l,1,25)$.

4.2. Simulation Results with the Dimension of Phenotypes Given by K = 3

Following the similar simulation set-up as those described in simulation studies in the text, we consider the continuous traits with the dimension of phenotypes given by K = 3. Preciously, the error terms $\boldsymbol{\varepsilon}_i = (\varepsilon_{i11}, \varepsilon_{i21}, \varepsilon_{i31}, \varepsilon_{i12}, \varepsilon_{i22}, \varepsilon_{i32}, \varepsilon_{i13}, \varepsilon_{i23}, \varepsilon_{i33})^T$ in equation (11) with K = 3 are assumed

to be from a multivariate normal distribution having a mean of zero, a within-in cluster correlation matrix (i.e., $\mathrm{Cor}(\varepsilon_{ijk}, \varepsilon_{ij'k})$) with diagonal entries of 1 and all off-diagonal entries of 0.2 and a subject-across-response correlation matrix (i.e., $\mathrm{Cor}(\varepsilon_{ijk}, \varepsilon_{ij'k'})$) with diagonal entries of 0.3 and all off-diagonal entries of 0.1. The empirical type I error rates based on fifty thousand replicates and the empirical power rates based on two thousand replicates are reported for all simulation results.

Empirical Type I Error Rates

Table S4 shows the empirical type I error rates of the seven competing methods, HoK, HoO, HeK, HeO, BT, mPK and mPB, with continuous traits based on the dimension of phenotypes given by $K = 3$. Table S4 shows that, in general, the seven competing methods have good performance on the empirical type I error rates with the unweighted marker-specific weight $w_l = Beta(m_l, 1, 1) = 1$ considered for variant *l* for continuous traits. On the other hand, the proposed method, HoK, and the existing method, mPK, have slightly high values of the empirical type I errors at the nominal level of 0.0001, when the marker-specific weight is considered for $w_l = Beta(m_l, 1, 25)$ for variant *l*.

In summary, the seven competing methods based on the unweighted marker-specific weight $w_l = Beta(m_l, 1, 1) = 1$ have more robust control than that based on weighted marker-

specific weight $w_l = Beta(m_l, 1, 25)$ in terms of the empirical type I errors for continuous traits, when the dimension of phenotypes is given by K = 3.

Table S4: Empirical type I errors of the seven competing methods with continuous traits based on the dimension of phenotypes given by K = 3.

Marker-specific weight (w_l)	Nominal level	Working correlation	Method						
			HoK[3]	HoO	HeK	HeO	BT	mPK[4]	mPB
Unweighted marker-specific weight[1]	0.05	U/U[2]	0.04854	0.05128	0.05068	0.05332	0.04950	0.04414	0.04820
		E/E	0.04860	0.05122	0.05114	0.05318	0.04980		
	0.01	U/U	0.00960	0.00990	0.00998	0.01040	0.01032	0.00858	0.00972
		E/E	0.00966	0.00978	0.00988	0.01016	0.01044		
	0.001	U/U	0.00068	0.00064	0.00074	0.00072	0.00082	0.00086	0.00098
		E/E	0.00066	0.00064	0.00068	0.00072	0.00088		
	0.0001	U/U	0.00006	0.00008	0.00008	0.00012	0.00010	0.00014	0.00008
		E/E	0.00006	0.00010	0.00008	0.00012	0.00010		
Weighted marker-specific weight	0.05	U/U	0.05200	0.05008	0.05074	0.04942	0.04696	0.04666	0.04614
		E/E	0.05200	0.05052	0.05068	0.04946	0.04686		
	0.01	U/U	0.00990	0.00932	0.01030	0.01004	0.00914	0.00998	0.00990
		E/E	0.00986	0.00932	0.01010	0.01032	0.00912		
	0.001	U/U	0.00098	0.00106	0.00114	0.00094	0.00090	0.00108	0.00106
		E/E	0.00094	0.00102	0.00118	0.00088	0.00094		
	0.0001	U/U	**0.00016**	0.00012	0.00012	0.00008	0.00010	**0.00018**	0.00014
		E/E	**0.00018**	0.00012	0.00012	0.00008	0.00008		

[1]The unweighted marker-specific weight is given by $w_l = Beta(m_l, 1, 1) = 1$; the weighted marker-specific weight is given by $w_l = Beta(m_l, 1, 25)$.

[2]U/U represents the structures of the working within-cluster and multivariate-response correlation matrices considered by the unstructured structures; E/E represents the structures of the working within-cluster and multivariate-response correlation matrices considered by the exchangeable structures.

[3]HoK, HoO, HeK, HeO and BT are our proposed methods.

[4]mPK and mPB are executed by the R package *MultiSKAT* [8].

Empirical Power

Figure S4 shows the empirical power rates of the seven competing methods, HoK, HoO, HeK, HeO, BT, mPK and mPB, with continuous traits based on the dimension of phenotypes given by $K = 3$, when the working within-cluster and multivariate-response correlation matrices of the proposed methods, HoK, HeK and BT, are considered to be exchangeable. From Figure S4, we observe that, in general, the empirical power rates based on the higher dimension of phenotypes in Figure S4 are larger than that based on the lower dimension of phenotypes in Figure 1, when continuous traits are considered. However, we note that when the genetic effects on the different phenotypes are homogeneous (i.e., $\boldsymbol{\beta}_1 = \boldsymbol{\beta}_2 = \boldsymbol{\beta}_3$), the empirical power rates of the proposed method, HeK, based on higher dimensions of phenotypes don't have an increasing trend, because the higher dimension of the phenotype causes the test statistic under the null hypothesis with a higher degree of freedom.

In summary, our simulation results show that, in general, the empirical power rates based on the higher dimension of phenotypes are larger than that based on the lower dimension of phenotypes, when continuous traits are considered.

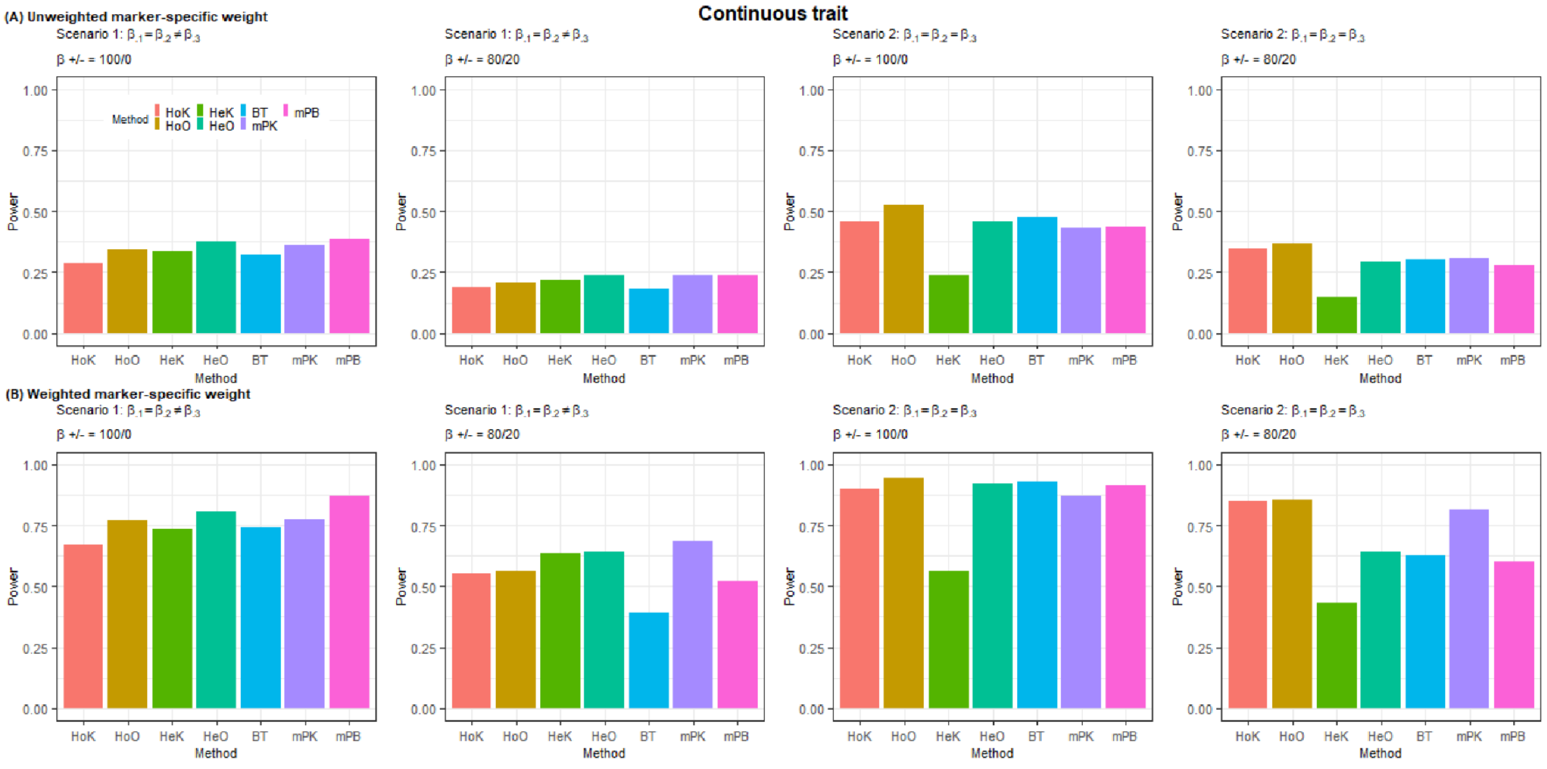

Figure S4: Power comparisons of the seven competing methods with continuous traits based on the dimension of phenotypes given by $K = 3$ for each scenario at the nominal level of 0.001. (A) Unweighted marker-specific weight: $w_l = Beta(m_l, 1, 1) = 1$. (B) Weighted marker-specific weight: $w_l = Beta(m_l, 1, 25)$.

Appendix S5. Additional Simulation Studies for Binary Traits

In this section we present addition simlation results to futher examine the performance of the proposed methods with binary traits.

5.1. Simulation Results with Slightly Higher Correlations of Phenotype Traits

Following the same simulation set-up as those described in simulation studies in the text, we consider the slightly higher correlational relationships for binary traits. Preciously, the binary traits $\boldsymbol{y}_i$ in equation (12) are generated by the R package *BinNor* [9] based on a within-in cluster correlation matrix (i.e., $\text{Cor}(y_{ijk}, y_{ij'k})$) with diagonal entries of 1 and all off-diagonal entries of 0.25 and a subject-across-response correlation matrix (i.e., $\text{Cor}(y_{ijk}, y_{ij'k'})$) with diagonal entries of 0.3 and all off-diagonal entries of 0.15. Because implementing the proposed tests, HoK, HoO, HeK, HeO, and BT, based on the binary traits needs high computational costs, the empirical type I error rates based on 15,000 replicates and the empirical power rates based on 2,000 replicates are reported for all simulation results in order to save time.

Empirical Type I Error Rates

Table S5 exhibits the empirical type I error rates of the proposed methods, HoK, HoO, HeK, HeO, and BT, with binary traits based on slightly higher correlations between phenotypes. Table

S5 displays that, in general, the five proposed methods can control the empirical type I error rates with the marker-specific weight considered for $w_l = Beta(m_l, 1, 1) = 1$ or $w_l = Beta(m_l, 1, 25)$ for variant *l* for binary traits. However, we observe that the proposed test, HoO, has inflated type I errors at the nominal level of 0.0001 with the weighted marker-specific weight $w_l = Beta(m_l, 1, 25)$ for variant *l*.

In summary, the five proposed methods based on the unweighted marker-specific weight $w_l = Beta(m_l, 1, 1) = 1$ have better performance than that based on the weighted marker-specific weight $w_l = Beta(m_l, 1, 25)$ in terms of the empirical type I errors for binary traits, when these traits have slightly higher correlations among one another.

Table S5: Empirical type I errors of the five competing methods with binary traits based on slightly higher correlations between phenotypes.

Marker-specific weight (w_l)	Nominal level	Working correlation	Method				
			HoK[3]	HoO	HeK	HeO	BT
Unweighted marker-specific weight[1]	0.05	U/U[2]	0.05033	0.05240	0.05480	0.05453	0.05153
		E/E	0.05067	0.05280	0.05500	0.05433	0.05107
	0.01	U/U	0.00900	0.00953	0.00987	0.00987	0.01007
		E/E	0.00913	0.00967	0.00993	0.00993	0.00993
	0.001	U/U	0.00053	0.00047	0.00107	0.00060	0.00060
		E/E	0.00060	0.00060	0.00113	0.00053	0.00067
	0.0001	U/U	0.00007	0.00007	0.00007	0.00000	0.00007
		E/E	0.00007	0.00007	0.00007	0.00000	0.00007
Weighted marker-specific weight	0.05	U/U	0.03920	0.03793	0.04987	0.05040	0.03580
		E/E	0.03860	0.03820	0.05007	0.05000	0.03633
	0.01	U/U	0.00887	0.00893	0.01087	0.00900	0.00733
		E/E	0.00900	0.00900	0.01100	0.00900	0.00720
	0.001	U/U	0.00093	0.00060	0.00107	0.00060	0.00033
		E/E	0.00100	0.00060	0.00113	0.00060	0.00033
	0.0001	U/U	0.00013	**0.00020**	0.00013	0.00007	0.00007
		E/E	0.00013	**0.00020**	0.00013	0.00007	0.00007

[1]The unweighted marker-specific weight is given by $w_l = Beta(m_l, 1, 1) = 1$; the weighted marker-specific weight is given by $w_l = Beta(m_l, 1, 25)$.

[2]U/U represents the structures of the working within-cluster and multivariate-response correlation matrices considered by the unstructured structures; E/E represents the structures of the working within-cluster and multivariate-response correlation matrices considered by the exchangeable structures.

[3]HoK, HoO, HeK, HeO and BT are our proposed methods.

Empirical Power

Figure S5 shows the empirical power rates of the five proposed methods, HoK, HoO, HeK, HeO and BT, with binary traits based on slightly higher correlations between phenotypes, when the working within-cluster and multivariate-response correlation matrices of the proposed methods,

HoK, HeK and BT, are considered to be exchangeable. In comparison with the empirical power rates presented in Figure 2 and Figure S5, the empirical power rates based on the slightly higher correlations of phenotypes in Figure S5 are similar to that based on the slightly lower correlations of phenotypes in Figure 2. The reason is that the value of the binary trait is 0 or 1, and the number of the one value is fewer in contrast with the number of the zero value. Moreover, as mentioned for continuous traits, the pattern of the empirical power rates presented in Figure 2 is analogous to that in Figure S5, because they both have similar correlation structures of phenotypes.

In summary, our simulation results show that, in general, the empirical power rates based on the slightly higher correlations of phenotypes are similar to or equal to that based on the lower correlations of phenotypes, when binary traits are considered.

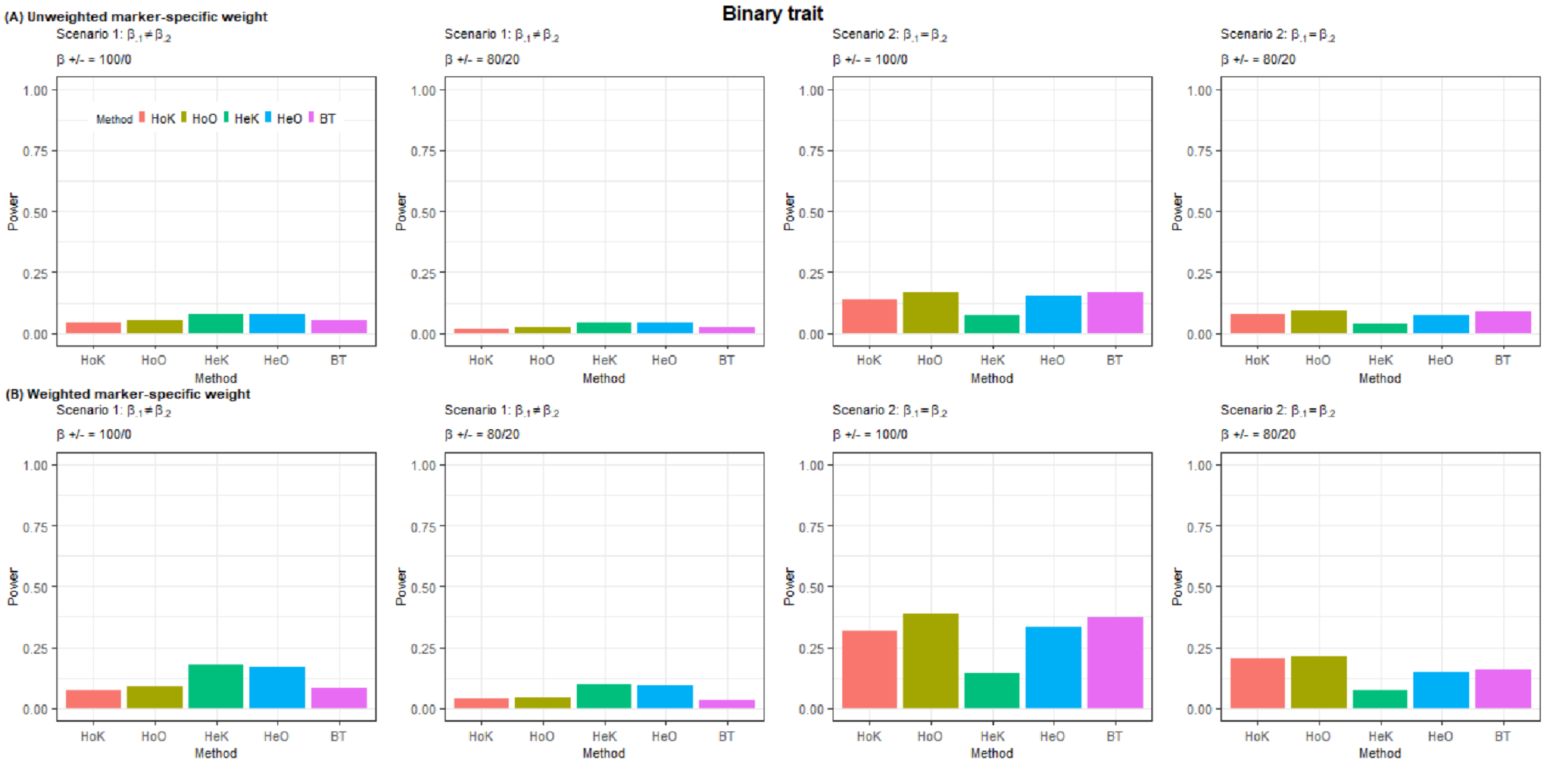

Figure S5: Power comparisons of the five competing methods with binary traits based on slightly higher correlations between phenotypes for each scenario at the nominal level of 0.001. (A) Unweighted marker-specific weight: $w_l = Beta(m_l, 1, 1) = 1$. (B) Weighted marker-specific weight: $w_l = Beta(m_l, 1, 25)$.

5.2. Simulation Results with the Dimension of Phenotypes Given by K = 3

Following the similar simulation set-up as those described in simulation studies in the text, we consider the binary traits with the dimension of phenotypes given by K = 3. Preciously, the binary traits $\boldsymbol{y}_i$ in equation (12) are generated by the R package *BinNor* [9] based on a within-in

cluster correlation matrix (i.e., $\mathrm{Cor}(y_{ijk}, y_{ij'k})$) with diagonal entries of 1 and all off-diagonal entries of 0.2 and a subject-across-response correlation matrix (i.e., $\mathrm{Cor}(y_{ijk}, y_{ij'k'})$) with diagonal entries of 0.3 and all off-diagonal entries of 0.1. Owing to binary traits with heavy computational requirements, the empirical type I error rates based on 15,000 replicates and the empirical power rates based on 2,000 replicates are reported for all simulation results in order to save time.

Empirical Type I Error Rates

Table S6 shows the empirical type I error rates of the proposed methods, HoK, HoO, HeK, HeO and BT, with binary traits based on the dimension of phenotypes given by $K = 3$. Table S6 shows that, in general, these proposed methods can appropriately control the empirical type I errors with the unweighted marker-specific weight $w_l = Beta(m_l, 1, 1) = 1$ or with the weighted marker-specific weight $w_l = Beta(m_l, 1, 25)$ for variant l for binary traits. However, the HeK, based on the unstructured structures of the working within-cluster and multivariate-response correlation matrices, has the empirical type I error rate inflation at the nominal level of 0.0001, when the marker-specific weight is considered for $w_l = Beta(m_l, 1, 25)$ for variant l.

In summary, the proposed methods, HoK, HoO, HeK, HeO and BT, based on the unweighted marker-specific weight $w_l = Beta(m_l, 1, 1) = 1$ have better performance than that based on weighted marker-specific weight $w_l = Beta(m_l, 1, 25)$ in terms of the empirical type I

errors for binary traits, when the dimension of phenotypes is given by $K = 3$.

Table S6: Empirical type I errors of the five competing methods with binary traits based on the dimension of phenotypes given by $K = 3$.

Marker-specific weight (w_l)	Nominal level	Working correlation	Method				
			HoK[3]	HoO	HeK	HeO	BT
Unweighted marker-specific weight[1]	0.05	U/U[2]	0.05040	0.05293	0.05273	0.05400	0.05147
		E/E	0.05040	0.05373	0.05240	0.05393	0.05133
	0.01	U/U	0.00993	0.00987	0.01013	0.01040	0.01027
		E/E	0.00993	0.00980	0.00967	0.01040	0.01047
	0.001	U/U	0.00067	0.00067	0.00120	0.00113	0.00093
		E/E	0.00080	0.00080	0.00133	0.00113	0.00100
	0.0001	U/U	0.00013	0.00007	0.00013	0.00013	0.00007
		E/E	0.00013	0.00007	0.00013	0.00013	0.00007
Weighted marker-specific weight	0.05	U/U	0.05280	0.05200	0.05380	0.05120	0.04687
		E/E	0.05313	0.05167	0.05380	0.05193	0.04687
	0.01	U/U	0.01087	0.01013	0.01087	0.00987	0.00880
		E/E	0.01120	0.01033	0.01087	0.00960	0.00887
	0.001	U/U	0.00080	0.00060	0.00107	0.00060	0.00067
		E/E	0.00087	0.00047	0.00100	0.00053	0.00067
	0.0001	U/U	0.00007	0.00007	**0.00020**	0.00013	0.00000
		E/E	0.00007	0.00007	0.00013	0.00013	0.00000

[1]The unweighted marker-specific weight is given by $w_l = Beta(m_l,1,1) = 1$; the weighted marker-specific weight is given by $w_l = Beta(m_l,1,25)$.

[2]U/U represents the structures of the working within-cluster and multivariate-response correlation matrices considered by the unstructured structures; E/E represents the structures of the working within-cluster and multivariate-response correlation matrices considered by the exchangeable structures.

[3]HoK, HoO, HeK, HeO and BT are our proposed methods.

Empirical Power

Figure S6 shows the empirical power rates of the proposed methods, HoK, HoO, HeK, HeO and

BT, with binary traits based on the dimension of phenotypes given by $K = 3$, when the working within-cluster and multivariate-response correlation matrices of the proposed methods, HoK, HeK and BT, are considered to be exchangeable. Compared with the empirical power rates in Figure 2 and Figure S6, we observe that, in general, the empirical power rates on the basis of the larger dimension of phenotypes in Figure S6 are bigger than that on the basis of the lower dimension of phenotypes in Figure 2, when the binary traits are considered. On the other hand, as mentioned for continuous traits, the higher dimension of the phenotype causes the HeK under the null hypothesis with a higher degree of freedom, which may cause that the empirical power rates of the HeK under the higher dimension of the phenotypes don't have an increasing trend, when the genetic effects on the different phenotypes are homogeneous (i.e., $\boldsymbol{\beta}_1 = \boldsymbol{\beta}_2 = \boldsymbol{\beta}_3$).

In summary, our simulation results show that, in general, the proposed methods, HoK, HoO, HeK, HeO and BT, based on the higher dimension of phenotypes can provide higher power rates for analyzing binary traits, in comparison with these methods based on the lower dimension of phenotypes.

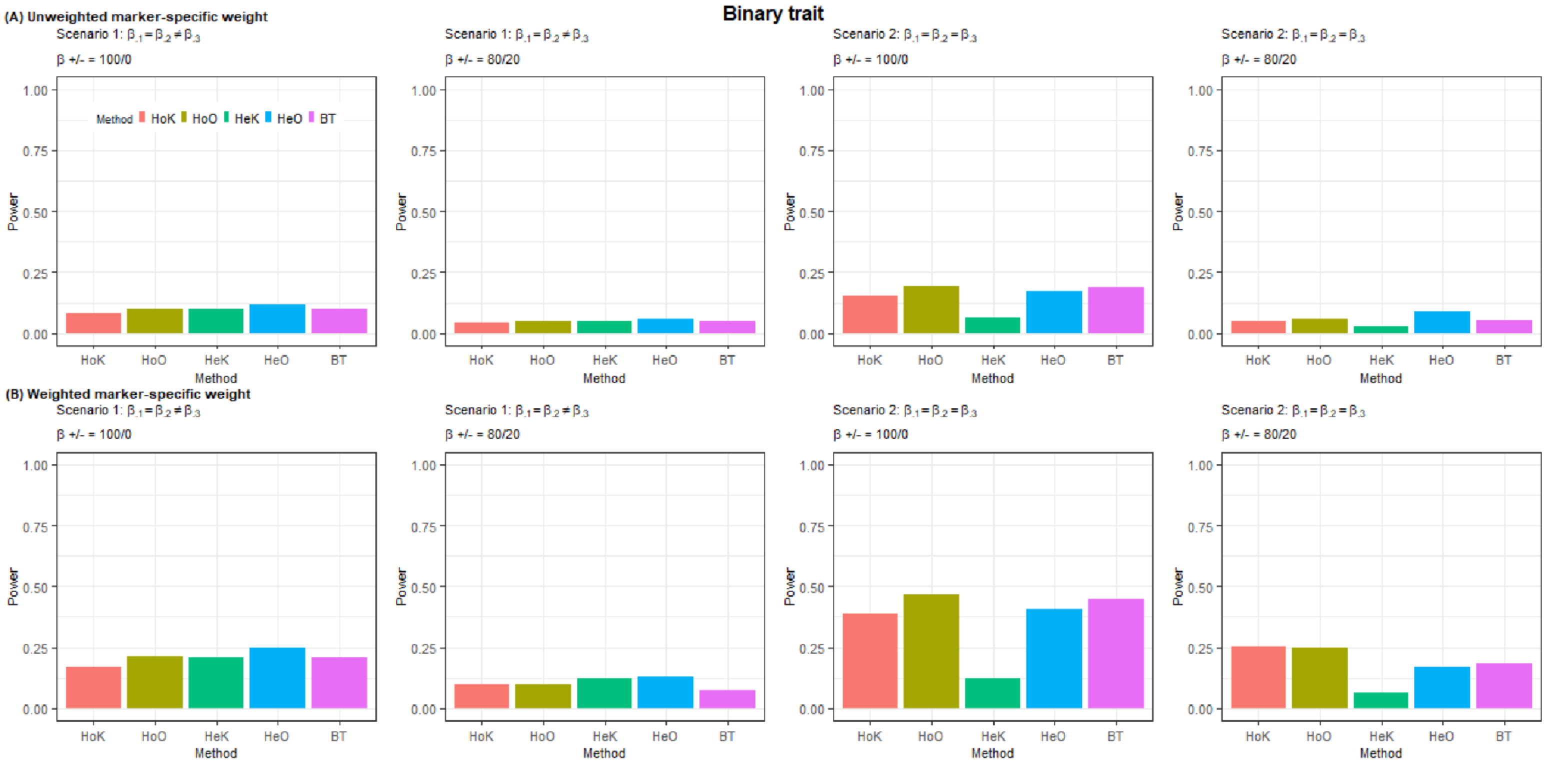

Figure S6: Power comparisons of the five competing methods with binary traits based on the dimension of phenotypes given by K = 3 for each scenario at the nominal level of 0.001. (A) Unweighted marker-specific weight: $w_l = Beta(m_l, 1, 1) = 1$. (B) Weighted marker-specific weight: $w_l = Beta(m_l, 1, 25)$.

Appendix S6. Limitation

To futher evaluate the performance of the empirical type I erors of the proposed methods for binary traits, we enhance the correlational relationship between phenotypes.

Following the same simulation set-up as those described in simulation studies in the text, the binary traits $\boldsymbol{y}_i$ in equation (12) are generated by the R package *BinNor* [9] based on a within-in cluster correlation matrix (i.e., $\mathrm{Cor}(y_{ijk}, y_{ij'k})$) with diagonal entries of 1 and all off-diagonal entries of 0.3 and a subject-across-response correlation matrix (i.e., $\mathrm{Cor}(y_{ijk}, y_{ij'k'})$) with diagonal entries of 0.3 and all off-diagonal entries of 0.2. The empirical type I error rates are carried out based on 15,000 simulation runs.

Table S7 shows the empirical type I rates of the five proposed methods, HoK, HoO, HeK, HeO and BT, with binary traits based on higher correlations between phenotypes. From Table S7, we observe that all proposed methods, HoK, HoO, HeK, HeO and BT, have good performance in terms of empirical type I error rates at the nominal of 0.001. On the other hand, we note that the proposed method HeK has the empirical type I error inflation at the nominal level of 0.0001 with the unweighted marker-specific weight $w_l = Beta(m_l, 1, 1)$ for variant *l*. Moreover, the proposed method HoK has the empirical type I error inflation at the nominal level of 0.0001 with the weighted marker-specific weight $w_l = Beta(m_l, 1, 25)$ for variant *l*. Compared with the empirical type I errors in Table 2, Table S5 and Table S7, we observe that these

proposed methods have more reasonable control in terms of the empirical type I errors for binary traits when the lower correlational relationships between the phenotypes are considered. Therefore, improving the proposed methods for more effectively analyzing the binary traits with higher correlations of phenotypes is the future work. This issue has been discussed in the Limitation section.

Table S7: Empirical type I errors of the five competing methods with binary traits based on higher correlations between phenotypes.

Marker-specific weight (w_l)	Nominal level	Working correlation	Method				
			HoK[3]	HoO	HeK	HeO	BT
Unweighted marker-specific weight[1]	0.05	U/U[2]	0.05047	0.05200	0.05360	0.05300	0.05220
		E/E	0.04993	0.05247	0.05373	0.05287	0.05220
	0.01	U/U	0.00893	0.00913	0.00987	0.00920	0.00940
		E/E	0.00880	0.00907	0.01007	0.00900	0.00947
	0.001	U/U	0.00060	0.00053	0.00100	0.00087	0.00060
		E/E	0.00067	0.00053	0.00093	0.00080	0.00067
	0.0001	U/U	0.00007	0.00013	**0.00020**	0.00013	0.00007
		E/E	0.00007	0.00013	**0.00020**	0.00013	0.00007
Weighted marker-specific weight	0.05	U/U	0.04880	0.04920	0.05193	0.04920	0.04733
		E/E	0.04833	0.04867	0.05207	0.04993	0.04727
	0.01	U/U	0.01113	0.01027	0.01040	0.01020	0.00847
		E/E	0.01087	0.01040	0.01033	0.01007	0.00840
	0.001	U/U	0.00133	0.00067	0.00107	0.00080	0.00067
		E/E	0.00133	0.00073	0.00100	0.00073	0.00067
	0.0001	U/U	**0.00020**	0.00013	0.00013	0.00007	0.00007
		E/E	**0.00020**	0.00013	0.00013	0.00007	0.00007

[1]The unweighted marker-specific weight is given by $w_l = Beta(m_l,1,1) = 1$; the weighted marker-specific weight is given by $w_l = Beta(m_l,1,25)$.

[2]U/U represents the structures of the working within-cluster and multivariate-response correlation matrices considered by the unstructured structures; E/E represents the structures of the working within-cluster and multivariate-response correlation matrices considered by the

exchangeable structures.

[3]HoK, HoO, HeK, HeO and BT are our proposed methods.

References

[1] D. J. Schaid, S. K. McDonnell, J. P. Sinnwell and S. N. Thibodeau, "Multiple genetic variant association testing by collapsing and kernel methods with pedigree or population structured data," *Genetic Epidemiology*, vol. 37, pp. 409-418, 2013.

[2] J. Yang, S. H. Lee, M. E. Goddard and P. M. Visscher, "GCTA: A tool for genome-wide complex trait analysis," *The American Journal of Human Genetics*, vol. 88, pp. 76-82, 2011.

[3] H. Chen, J. B. Meigs and J. Dupuis, "Sequence kernel association test for quantitative traits in family samples," *Genetic Epidemiology*, vol. 37, pp. 196-204, 2013.

[4] Y. Liu, S. Chen, Z. Li, A. C. Morrison, E. Boerwinkle and X. Lin, "ACAT: A fast and powerful p value combination method for rare-variant analysis in sequencing studies," *The American Journal of Human Genetics*, vol. 104, pp. 410–421, 2019.

[5] Y. Liu and J. Xiey, "Cauchy combination test: A powerful test with analytic p-value calculation under arbitrary dependency structures," *Journal of the American Statistical Association*, vol. 115, pp. 393-402, 2020.

[6] Z. R. McCaw, J. M. Lane, R. Saxena, S. Redline and X. Lin, "Operating characteristics of the rank-based inverse normal transformation for quantitative trait analysis in genome-wide association studies," *Biometrics*, pp. 10.1111/biom.13214, 2019.

[7] Z. R. McCaw, "Rank normal transformation omnibus test," *R package version 0.7.1*, 2019.

[8] D. Dutta, L. Scott, M. Boehnke and S. Lee, "Multi-SKAT: General framework to test for rare-variant association with multiple phenotypes," *Genetic Epidemiology*, vol. 43, pp. 4-23, 2019.

[9] A. Amatya, H. Demirtas and R. Gao, "Simultaneous generation of multivariate binary and normal variates," *R package version 2.3.2*, 2020.
